# Evolution of the Mutation Spectrum Across a Mammalian Phylogeny

**DOI:** 10.1093/molbev/msad213

**Published:** 2023-09-28

**Authors:** Annabel C Beichman, Jacqueline Robinson, Meixi Lin, Andrés Moreno-Estrada, Sergio Nigenda-Morales, Kelley Harris

**Affiliations:** Department of Genome Sciences, University of Washington, Seattle, WA, USA; Institute for Human Genetics, University of California, San Francisco, CA, USA; Department of Plant Biology, Carnegie Institution for Science, Stanford, CA, USA; National Laboratory of Genomics for Biodiversity, Advanced Genomics Unit (UGA-LANGEBIO), CINVESTAV, Irapuato, Mexico; Department of Biological Sciences, California State University, San Marcos, San Marcos, CA, USA; Department of Genome Sciences, University of Washington, Seattle, WA, USA; Herbold Computational Biology Program, Fred Hutchinson Cancer Center, Seattle, WA, USA

**Keywords:** mutation, mutation spectrum, mutagenesis, mammal, phylogenetic signal, reproductive age

## Abstract

Although evolutionary biologists have long theorized that variation in DNA repair efficacy might explain some of the diversity of lifespan and cancer incidence across species, we have little data on the variability of normal germline mutagenesis outside of humans. Here, we shed light on the spectrum and etiology of mutagenesis across mammals by quantifying mutational sequence context biases using polymorphism data from thirteen species of mice, apes, bears, wolves, and cetaceans. After normalizing the mutation spectrum for reference genome accessibility and *k*-mer content, we use the Mantel test to deduce that mutation spectrum divergence is highly correlated with genetic divergence between species, whereas life history traits like reproductive age are weaker predictors of mutation spectrum divergence. Potential bioinformatic confounders are only weakly related to a small set of mutation spectrum features. We find that clock-like mutational signatures previously inferred from human cancers cannot explain the phylogenetic signal exhibited by the mammalian mutation spectrum, despite the ability of these signatures to fit each species’ 3-mer spectrum with high cosine similarity. In contrast, parental aging signatures inferred from human de novo mutation data appear to explain much of the 1-mer spectrum's phylogenetic signal in combination with a novel mutational signature. We posit that future models purporting to explain the etiology of mammalian mutagenesis need to capture the fact that more closely related species have more similar mutation spectra; a model that fits each marginal spectrum with high cosine similarity is not guaranteed to capture this hierarchy of mutation spectrum variation among species.

## Introduction

Germline mutations likely arise from a mixture of DNA replication errors and chemical DNA damage (Lindahl and Wood 1999; Hoeijmakers 2001). Although the relative contributions of these endogenous and exogenous processes are unknown, the action of specific mutagens can sometimes be inferred by classifying mutations into a spectrum of measurable mutation types, for example, single nucleotide polymorphisms (SNPs) occurring in different 3-mer contexts (Hwang and Green 2004; Alexandrov et al. 2013). Studies of somatic mutations in cancer have revealed that exogenous mutagens and DNA repair deficiencies can dramatically affect the mutation spectrum in a way that is informative about the biology of cancer and its likely susceptibility to chemotherapies (Nik-Zainal et al. 2012). Many of the same mutational processes also affect normal tissues and provide insights into mechanisms of aging (Martincorena et al. 2015; Cagan et al. 2022).

Germline mutation spectra tend to be less variable than somatic mutation spectra—although mutational signature analysis methods have uncovered scores of different mutational processes that operate in different tumor genomes, these methods infer that germline mutations are a relatively homogeneous mixture of just 2 to 4 processes (Rahbari et al. 2016; Moore et al. 2021). However, more sensitive analysis methods have revealed the existence of subtle but robust differences among the mutation spectra of human populations (Harris 2015; Harris and Pritchard 2017; Mathieson and Reich 2017; Narasimhan et al. 2017; Moore et al. 2021; Gao et al. 2023). Some of these differences reflect the aging of parental gametes; for example, children born to older mothers tend to have more C > G de novo mutations (Goldmann et al. 2016; Wong et al. 2016; Jónsson et al. 2017). Measurements of germline mutation accumulation patterns are beginning to overturn long-held theories about the biology of reproduction, including the assumption that most genetic variation stems from DNA replication errors in the adult testis (Gao et al. 2019; Wu et al. 2020; Seplyarskiy and Sunyaev 2021; Hahn et al. 2023). If most mutations were the result of replication errors, then the number of mutations present in maternally inherited DNA should not scale with maternal age, yet de novo mutation data have revealed that mutations accumulate every year in eggs as well as spermatocytes (Jónsson et al. 2017; Goldmann et al. 2018; Gao et al. 2019). These maternal germline mutations that accumulate with age must have an etiology that is not replication-dependent, which calls into question the assumption that mutations accumulating in dividing cells are the result of cell division errors rather than DNA damage.

One source of information about germline mutagenesis is genetic variation: polymorphisms are relics of mutations that occurred many generations ago. Polymorphisms’ mutation spectra can be complicated to interpret because of perturbations introduced by natural selection and biased gene conversion (Duret and Galtier 2009; Ratnakumar et al. 2010; Vollger et al. 2023), but they suggest that many species and populations have distinct mutation spectra (Moorjani et al. 2016; Harris and Pritchard 2017; Mathieson and Reich 2017; Dumont 2019; Jiang et al. 2021; Goldberg and Harris 2022; Sasani et al. 2022; Bloom et al. 2023) and that these differences generally do not fit the classical profile of biased gene conversion (Harris and Pritchard 2017; Gao et al. 2023). Mutation spectrum variation is generally inferred from polymorphisms in nonconserved, noncoding genomic regions, meaning that natural selection is not likely to be the driving force behind these differences.

One pattern that has been qualitatively noted in humans and other great apes is that the mutation spectrum appears to be a phenotype with phylogenetic signal (DeWitt et al. 2021; Goldberg and Harris 2022), meaning that more distantly related lineages generally have less similar mutation spectra than more closely related lineages. This pattern is consistent with the hypothesis that the mutation spectrum is a genetically determined phenotype that evolves over time due to the emergence of new mutator alleles (Sturtevant 1937; Lynch 2010; Sung et al. 2012; Lynch et al. 2016) that each perturb different DNA repair pathways and tend to act in different sequence contexts. Mutator variants have been identified in human families (Robinson et al. 2021; Kaplanis et al. 2022) as well as certain populations of yeast, mice, and primates (Jiang et al. 2021; Sasani et al. 2022; Stendahl et al. 2023), but these variants can only explain a small proportion of the mutation spectrum variation that exists within these species. It is unclear whether the remaining variation was created by undiscovered mutators versus environmental mutagens or changes in the timing of reproduction, which might also create phylogenetic signal under certain circumstances (Thomas et al. 2018; Coll Macià et al. 2021; Wang et al. 2022; Wang et al. 2023).

Life history traits such as generation time have a clear impact on the germline mutation rate and spectrum (as well as the somatic mutation spectrum) (Risch et al. 1987; Sayres et al. 2011; Bromham et al. 2015; Cagan et al. 2022). Body size and longevity may also affect germline mutagenesis by incentivizing evolution of better DNA repair to avoid cancer growth (Nabholz et al. 2008; Caulin and Maley 2011; Abegglen et al. 2015; Vazquez and Lynch 2021); in rockfish, longevity appears to be correlated with the rate of CpG transition mutations (Kolora et al. 2021). A large recent study of vertebrate de novo mutations found support for the idea that generation time affects the mutation rate, though interestingly it found no support for the impact of body size (Bergeron et al. 2023). To better understand how genetics, environment, and age interact to shape the accumulation of mutations in the germline, more standardized mutation data from a variety of taxa will be needed.

In this study, we use publicly available whole-genome polymorphism data to study mutation spectrum evolution over a phylogeny that spans rodents, primates, cetaceans, and carnivorans. We generate mutation spectra from each species using a pipeline that is designed to minimize variation caused by reference genome composition, sample size, genome accessibility, and population history. Since bioinformatic batch effects are a significant obstacle to the reanalysis of data from multiple studies that were generated at different times using different technologies under different budgetary constraints, we explore the apparent dependence of the mutation spectrum on confounders, including genome assembly quality and resequencing read coverage (Taub et al. 2010; Tom et al. 2017; Leigh et al. 2018; Anderson-Trocmé et al. 2020). We then use these data to explore how the mutation spectrum might evolve as a function of biological variables like reproductive life history, testing the predictions of several key hypotheses about the origin and evolution of germline mutations.

## Results

### Standardizing the Mutation Spectrum for Genome Composition and Genetic Diversity

**Fig. 1.**
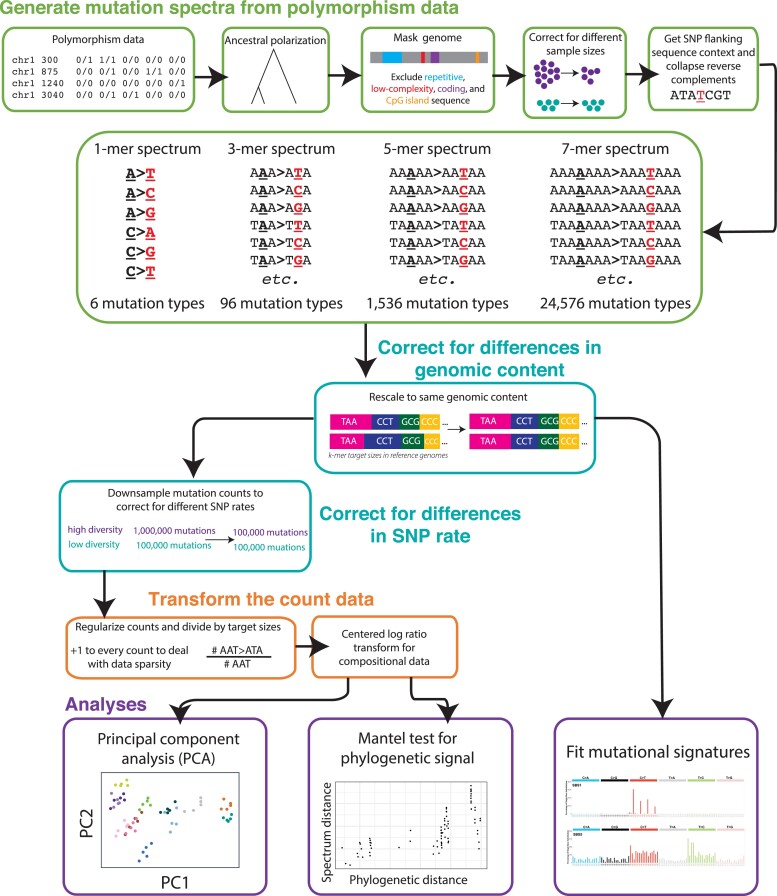
Analysis workflow. Our approach for comparing mutation spectra between species. Details in Methods and SI Methods. Mutational signature images from the Catalogue of Somatic Mutations in Cancer (COSMIC) database.

**Fig. 2.**
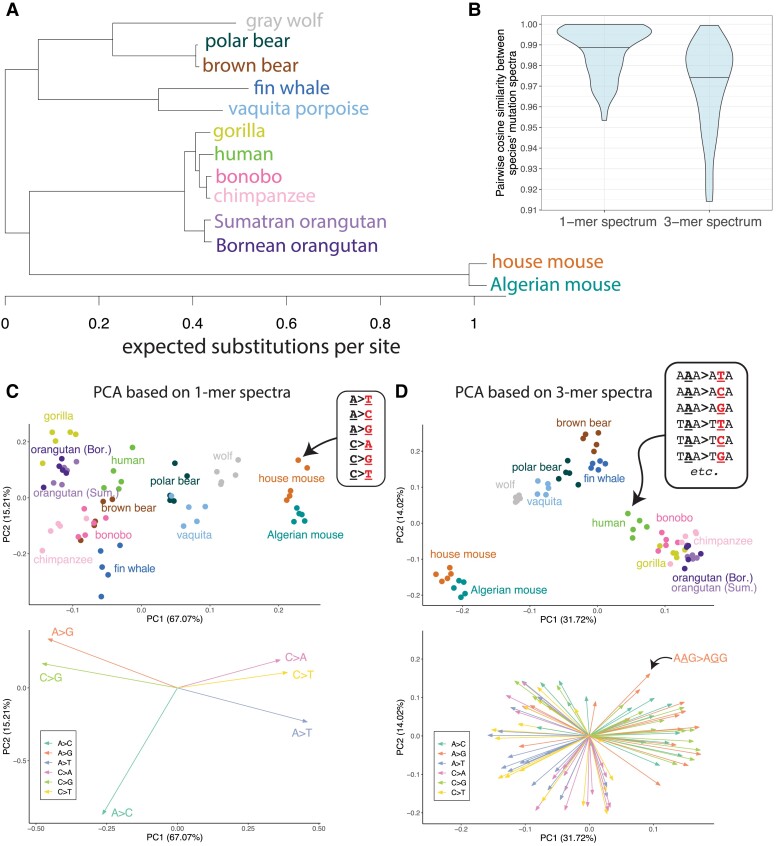
Principal components of 1-mer and 3-mer mutation spectrum variation reflect phylogenetic relationships among species. a) *RAxML* tree from Upham et al. (2019), restricted to species included in our study. Branch lengths represent the expected substitutions per site in Upham et al.'s 31-gene sequence alignment. b) Distributions of cosine similarities between 1-mer and 3-mer mutation spectra for every pair of species in our dataset (supplementary Table S1, Supplementary Material online). Horizontal lines denote the median. c) PCA of 1-mer mutation spectra. Each point represents a single individual's 1-mer mutation spectrum. Points are colored and labeled according to species membership (supplementary Table S1, Supplementary Material online). To avoid oversampling any species, 5 representative individuals were chosen at random from each species. SNPs were rescaled to the same genomic content across species and multinomial-downsampled to the minimum number of SNPs observed across all individuals. The resulting mutation spectra were centered log-ratio (CLR) transformed as described in the SI Methods. PCA loadings are shown in the panel below, colored and labeled by mutation type (dark green: A > C; orange: A > G; purple: A > T; pink: C > A; light green: C > G; yellow: C > T). d) PCA of 3-mer spectra constructed as in (b). 3-mer mutation type loadings in the lower panel are colored by their central mutation types (e.g. A**A**G > A**G**G is labeled for illustration, and is colored orange as it is a type of A > G mutation). Plots including PC3 are shown in supplementary Fig. S2, Supplementary Material online. PCAs of isometric log-ratio (ILR) transformed spectra, which qualitatively resemble the CLR transformed spectra, are shown in supplementary Fig. S3, Supplementary Material online. Plots coloring/shaping points by sequencing platform/read length are in supplementary Fig. S4, Supplementary Material online.

We estimated 1-mer, 3-mer, 5-mer, and 7-mer mutation spectra (Fig. 1) using SNPs annotated as high quality in whole-genome sequence data sampled from 6 primate species (human, chimpanzee, bonobo, gorilla, Sumatran orangutan, and Bornean orangutan), 2 rodents (house mouse and Algerian mouse), 2 cetaceans (fin whale and vaquita porpoise), and 3 carnivorans (brown bear, polar bear, and gray wolf) (Miller et al. 2012; Cahill et al. 2013; Prado-Martinez et al. 2013; Liu et al. 2014; Harr et al. 2016; Benazzo et al. 2017; Barlow et al. 2018; Byrska-Bishop et al. 2022; Morrill et al. 2022; Robinson et al. 2022; Nigenda-Morales et al. 2023) (Fig. 2a, supplementary Table S1, Supplementary Material online). These species span 100 million years of mammalian evolution (supplementary Fig. S1, Supplementary Material online). They also vary considerably in body size (from 20 g to >40,000 kg), reproductive age (140 d to 23 yr), lifespan (4 to >80 yr), and environment, which are important variables that have the potential to influence DNA damage, repair, and replication.

To interpret the counts of each *k*-mer-based mutation type as proxies for context-dependent mutation rates, we developed a novel standardized pipeline to normalize these counts for accessible genome composition. We first excluded genomic regions where SNP calls are likely to be unreliable (low complexity regions, repeat regions, CpG Islands) as well as regions subject to strong purifying selection (genic regions and surrounding regulatory regions) (Fig. 1). We then transformed raw SNP counts to minimize differences between species caused by sample size, *k*-mer composition of the accessible part of the reference genome, and demographic history (Fig. 1). Finally, we transformed mutation spectrum distances via Aitchison's centered log-ratio (CLR) to eliminate spurious correlations that can affect vectors of compositional data (Pearson 1897; Aitchison 1986).

### Principal Component Analysis Reveals That Mutation Spectra Cluster by Phylogenetic Clade

After filtering and normalizing all species’ mutation spectra, we explored several strategies for measuring their similarity to one another. One metric commonly used to compare mutation spectra is cosine similarity (Kucab et al. 2019; Alexandrov et al. 2020). We observe high cosine similarity between pairs of species’ 1-mer and 3-mer spectra—roughly half the pairs have cosine similarity greater than 0.98, essentially identical by the standards used to compare cancer mutation spectra (Fig. 2b) (for comparison, 2 mutational processes with cosine similarity 0.9 are considered hard to distinguish from each other in Alexandrov et al. (2020)). However, since polymorphisms are much more numerous than somatic mutation counts derived from individual tumors, we hypothesized that high cosine similarity might mask differences that are robust and statistically significant. This hypothesis is supported by principal component analysis (PCA) of our normalized 1-mer and 3-mer spectra (Fig. 2c and d; supplementary Figs. S2 to S4, Supplementary Material online). The 3-mer spectrum reveals a particularly clear clustering of individuals by species and higher-order clade (Fig. 2d), echoing previous analyses of humans and great apes (Harris and Pritchard 2017; Goldberg and Harris 2022). Species and clade clustering are noisier in the 1-mer spectrum PCA (Fig. 2c), suggesting that sequence context is essential for resolving mutation spectrum differences among these species. Species do not cluster based on sequencing platform or read length, which is well distributed across the phylogeny (supplementary Fig. S4, Supplementary Material online). Notably, bears, wolves, vaquitas, and fin whales cluster together as per phylogenetic expectation (Fig. 2b), despite the fact that these species were all sequenced with different bioinformatics protocols and mapped to reference genomes of varying quality (supplementary SI Methods and Table S1, Supplementary Material online). We note that the mice, which are outliers on PC1, also have the greatest genetic distance to all other clades due to a long internal branch in the phylogeny (Fig. 2a) that is likely caused by the short murine generation time (Martin and Palumbi 1993).

### Testing for the Significance of Phylogenetic Signal

We used the Mantel test to quantify the correlation between phylogenetic distance and mutation spectrum divergence that is qualitatively seen in Fig. 2. This involves permuting the matrix of pairwise mutation spectrum distances to construct a well-calibrated null for assessing the significance of the spectrum distance's correlation with phylogenetic distance (Mantel 1967; Harmon and Glor 2010; Hardy and Pavoine 2012; Legendre and Legendre 2012). Phylogenetic branch lengths were calculated from a published *RAxML* tree (Upham et al. 2019) with branch lengths representing expected substitutions per site (Fig. 2a; ultrametric timetree in supplementary Fig. S1, Supplementary Material online). Since the divergence in a trait evolving under a Brownian motion model is expected to scale with the square root of cophenetic distance (Hardy and Pavoine 2012), we tested for a significant correlation between each mutation spectrum distance and the square root of the substitution rates that Upham et al. (2019) estimated using a multispecies sequence alignment.

**Fig. 3.**
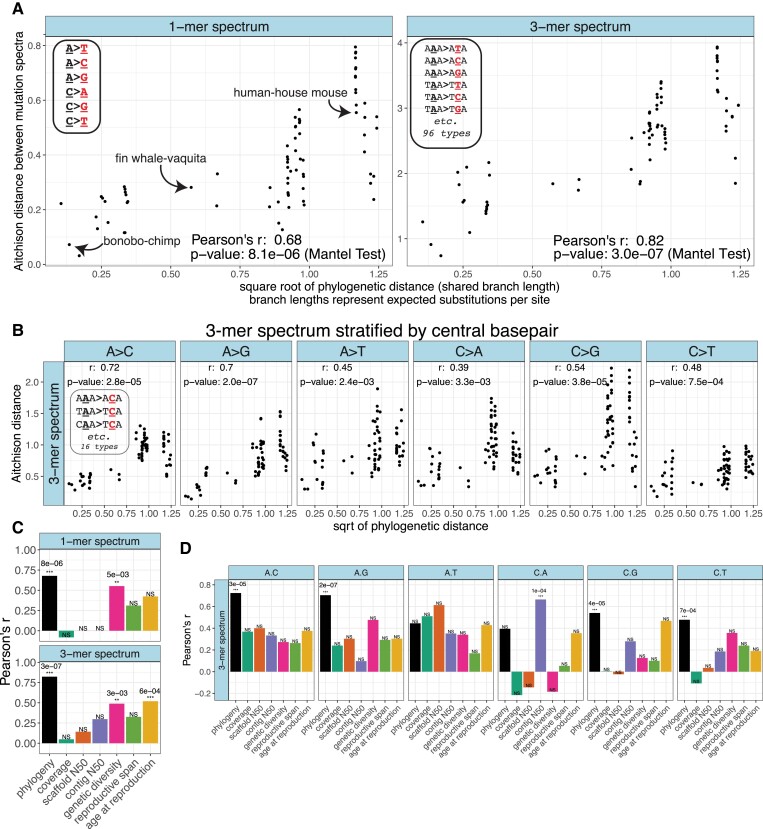
Mutation spectrum distance between species shows a phylogenetic signal. a) Correlation between pairwise mutation spectrum distance and the square root of phylogenetic distance between pairs of species. Distances reflect CLR-transformed 1-mer mutation spectra and 3-mer spectra for each species such that each point represents a single between-species comparison (e.g. human vs. house mouse). Note that unlike in Fig. 2, mutation spectra are calculated across all 5 sampled individuals per species, rather than per individual. *P*-values are based on the Mantel test with 9,999,999 permutations. A version of these plots with each point labeled is shown in supplementary Fig. S5, Supplementary Material online. b) Correlation between mutation spectrum distance and phylogenetic distance exists within the 3-mer subspectrum of each 1-mer mutation type (e.g. distances in the “A > C” column distances are calculated based on the 16 A > C 3-mers only). c) Values of Pearson's *r* for the correlations across species between technical and biological variables and mutation spectrum distance, calculated using the Mantel test with 99,999 permutations. The black “phylogeny” column represents the phylogenetic signal *P*-values reported in (a). “NS” (nonsignificant) denotes *P*-values that fell above a significance threshold Bonferroni-corrected for 7 tests (α = 0.007 = 0.05/7). *P*-values below this threshold are denoted using “**” if < 0.007 but > 0.001, and “***” if < 0.001, and the specific *P*-values are written above the corresponding column. d) Values of Pearson's *r* for the correlation between different variables and the 3-mer spectrum when it is stratified by central 1-mer type (as in (B)). *P*-values from Mantel tests with 99,999 permutations. The black “phylogeny” columns represent the *r*-values in (b). NS (nonsignificant) designates *P*-values that fall above a significance threshold Bonferroni-corrected for 42 tests: 7 confounders for each of 6 mutation types (α = 0.05/(6*7) = 0.001). *P*-values below this threshold are denoted with “***” and the specific *P*-value is written above the corresponding column. Note that when carrying out the Mantel test for these potential confounders we used fewer permutations than in (a) since no confounding variable reached the minimum *P*-value for 99,999 permutations (1e−5) that would have required additional permutations. Phylogenetically aware Mantel results for panels C and D are in supplementary Fig. S14, Supplementary Material online.

Using a Mantel Test with 9,999,999 permutations, we found that both the 6-dimensional 1-mer mutation spectrum and the 96-dimensional 3-mer spectrum exhibited a significant phylogenetic signal (*r* = 0.68, *P* < 8e−6 and *r* = 0.82, *P* < 3e−7; respectively) (Fig. 3a, supplementary Fig. S5, Supplementary Material online for labeled comparisons). This phylogenetic signal appears robust to many analysis variations, including using an ultrametric phylogenetic tree, substituting cosine distance or isometric log-ratio (ILR) distance for the CLR distance (Egozcue et al. 2003), and “folding” the mutation spectrum to remove any effects of ancestral allele misidentification (supplementary Figs. S6 to S9, Supplementary Material online). Due to their high mutation rates, CpG > TpG mutations are sometimes censored from polymorphism-based spectra or separated out as their own mutation class (Gao et al. 2023; Wang et al. 2023), and we find that neither of these choices appreciably reduces the phylogenetic signal (supplementary Fig. S10, Supplementary Material online).

We additionally tested for the presence of phylogenetic signal using the *K_mult_* statistic (Adams 2014; Adams and Collyer 2018), a multivariate version of Blomberg's *K* (Blomberg et al. 2003). We found significant phylogenetic signal at both the 1-mer and 3-mer levels (1-mer: *K_mult_* = 0.31, *P* < 0.001; 3-mer: *K_mult_* = 0.26, *P <* 0.001; 999 permutations) (supplementary Fig. S11, Supplementary Material online). The values of *K_mult_* were less than 1 (1 being the expected value under Brownian motion). This deviation of the Brownian motion expectation is typical of most assayed multivariate traits and could be due to noise or natural selection. Another possibility for *K_mult_* being less than 1 is that the number of independently varying mutation spectrum components may be much smaller than the dimensionality of the full *k*-mer mutation spectrum (Adams and Collyer 2019), as seen in mutational signature decompositions where a relatively small number of mutational processes explain mutation spectrum variability across samples.

### Mutation Spectrum Divergence Is Not Consistent With the GC-biased Signature of Gene Conversion

One potential contributor to phylogenetic divergence between mutation spectra is GC-biased gene conversion (gBGC), a process that drives mutations from A/T to G/C to rise in frequency over time while driving mutations from G/C to A/T to decline in frequency (Duret and Galtier 2009). Species with the highest effective population sizes are expected to experience the strongest gBGC, leading to a well-understood distortion of the mutation spectrum. However, gBGC is not expected to affect C > G or A > T mutations, and it is not known to affect the *k*-mer sequence composition within each 1-mer mutation class.

When we performed Mantel tests on the spectrum of 3-mer mutation types partitioned into categories that experience different modalities of gBGC-induced selection (neutral A > T and C > G; negatively selected C > A and C > T, and positively selected A > C, A > G), we found highly significant phylogenetic signal within each category, notably including the gBGC-neutral (A > T + C > G) category (supplementary Fig. S12, Supplementary Material online; *r* = 0.79, *P* < 5e−6). We then partitioned 3-mers by 1-mer mutation class to generate “subspectra” and still found significant phylogenetic signal, with Mantel test *P*-values ranging from 3.3e-3 (for C > A 3-mers) to 2e-7 (for A > G 3-mers; all less than the Bonferroni-corrected threshold α = 0.05/6 = 0.0083 appropriate for a set of 6 tests) (Fig. 3B). This implies that gBGC cannot be the primary force that causes the mutation spectrum to have phylogenetic signal.

### Differences in Bioinformatic Data Quality Are Unlikely to Explain the Observed Mutation Spectrum Differences Among Species

A potential caveat to the above results is that if data quality and bioinformatic processing tend to be more similar among more closely related species, this could create the false appearance of a correlation between mutation spectrum similarity and phylogenetic relatedness. There are several reassuring indications that our dataset does not have this property: for example, the best quality chromosomal genome assemblies by several metrics are human, mouse, and vaquita (supplementary Table S1, Supplementary Material online), species which are not closely related, and the oldest-generated datasets [the great apes (published in 2013), mice (2016), and bears (2012 to 2018)] are likewise dispersed across the phylogeny.

To formally test whether technical artifacts are phylogenetically distributed across our dataset, we used the Mantel test to measure the correlation of phylogenetic distance with 3 technical variables: average sequencing coverage, reference genome scaffold N50, and reference genome contig N50. Differences between species’ scaffold N50 and sequence coverage showed no significant correlation with phylogenetic distance (supplementary Fig. S13, Supplementary Material online). Differences in contig N50 (the relative contiguity of contigs before scaffolding) showed a moderate phylogenetic signal (Pearson's *r* = 0.42, *P*-value < 0.007, Mantel test with 99,999 permutations) (supplementary Fig. S13, Supplementary Material online), but this correlation coefficient and *P*-value are more modest than those of the correlation between mutation spectrum distance and phylogenetic distance (Fig. 3a). While technical confounders may explain a small portion of the phylogenetic signal in the dataset, they do not appear sufficient to explain the results shown in Fig. 3a.

To more directly assess what role (if any) these technical confounders may play in causing differences between our species’ mutation spectra, we directly tested each technical confounder for correlation with mutation spectrum distance (Mantel test with 99,999 permutations) (Fig. 3c). Our aim here was to test whether any of these measurements explain mutation spectrum divergence *better* than the phylogeny does. We found that differences in contig N50, scaffold N50, and sequence coverage between species are *not* significantly correlated with 1-mer and 3-mer mutation spectrum distance after correction for multiple testing (Fig. 3c). Our result indicates that these confounders cannot be responsible for the differences between mutation spectra we observe, though any correlations between the mutation spectrum and these technical measurements could result from a shared phylogenetic signal. We obtain qualitatively similar results using a phylogenetically aware Mantel test that asks whether each technical covariate explains additional mutation spectrum divergence on top of what is explained by the phylogeny (supplementary Fig. S14, Supplementary Material online).

When we look at the 3-mer subspectra of individual 1-mer mutations (particularly A > T and C > A 3-mers), it is less consistently clear that phylogeny explains subspectrum variation better than technical factors (Fig. 3d). After stratifying the spectra by central basepair, we observed that contig N50 is more significantly correlated with differences in the C > A 3-mer spectrum than phylogenetic distance is (*r* = 0.67, *P* < 1.1e−4 for the correlation between C > A 3-mers and contig N50 compared to *r* = 0.39, *P* < 3.3e−3 for C > A 3-mers and phylogenetic distance; Fig. 3d, supplementary Fig. S14, Supplementary Material online for phylogenetically aware Mantel results). Scaffold N50 is more correlated with A > T 3-mer spectrum distances than phylogenetic distance is (*r* = 0.6, *P* < 2.1e-3 for A > T 3-mers and scaffold N50 compared to *r* = 0.45, *P* < 2.4e−3 for A > T 3-mers and phylogenetic distance), though neither correlation passes the significance threshold after correction for multiple tests (α = 1e−3). However, after correcting for multiple testing, the phylogeny is the only significant covariate with 3-mer mutation spectrum divergence within each of the remaining 4 mutation types (A > C, A > G, C > G, and C > T).

### Differences in Reproductive Age and Effective Population Size Are Not the Primary Drivers of Mutational Phylogenetic Signal

Recent studies of human and animal de novo mutagenesis have found that the mutation rate and spectrum depend on age at reproduction (Goldmann et al. 2016; Wong et al. 2016; Jónsson et al. 2017; Thomas et al. 2018; Bergeron et al. 2023). Motivated by this, we performed additional tests to calculate the correlation of mutation spectrum divergence with maximum reproductive lifespan and age at first reproduction (Jones et al. 2009; Pacifici et al. 2013). We also measured the correlation between mutation spectrum distance and the genetic diversity metric Watterson's *θ*, since diversity is strongly correlated with the strength of gBGC. We expected these biological confounders to be at least partially phylogenetically distributed across our dataset, and so first tested each variable for a significant phylogenetic signal (supplementary Fig. S15, Supplementary Material online). Age at first reproduction exhibited no significant phylogenetic signal in this dataset after correction for multiple testing (Pearson's *r* = 0.32, *P* < 0.024, Bonferroni α = 0.008 as appropriate for 6 tests for phylogenetic signal across the 6 confounders, supplementary Fig. S15, Supplementary Material online), but reproductive lifespan and Watterson's *θ* each exhibited moderate phylogenetic signal (Pearson's *r* = 0.40, *P* < 0.005; *r* = 0.6, *P* < 0.0013, respectively) (supplementary Fig. S15, Supplementary Material online).

To determine what impact these biological variables may have in shaping mutation spectrum distances across our species, we then directly correlated each of them with pairwise mutation spectrum distances (Fig. 3c). Age at first reproduction is significantly correlated with differences in the 3-mer spectrum after correction for multiple testing (age at first reproduction: *r* = 0.52, *P* < 0.00057, Bonferroni α = 0.007 as appropriate for a set of 7 tests for correlation with different biological and technical variables) (Fig. 3c), but the correlation of phylogenetic distance with the 3-mer mutation spectrum is stronger (*r* = 0.82, *P* < 3e−7; Fig. 3a). Correlation between reproductive lifespan and the 3-mer mutation spectrum falls just short of the Bonferroni-corrected threshold (*r* = 0.33, *P* > 0.0072, Bonferroni α = 0.007) (Fig. 3c). Both age at first reproduction and reproductive lifespan appear significantly correlated with the 3-mer spectrum once phylogenetic relationships are accounted for using a phylogenetically aware version of the Mantel test (*P* < 8 × 10^−5^ and *P* < 0.004, respectively), indicating that it is unlikely that these relationships are driven by shared phylogenetic signal and may instead reflect a role of generation time in shaping mutation spectrum patterns between species (supplementary Fig. S14, Supplementary Material online). The correlation between age at first reproduction and the 1-mer spectrum falls just above the significance threshold after correction for multiple testing (*r* = 0.42, *P* > 0.0076, Bonferroni α = 0.007) (Fig. 3c), suggesting that it is important to consider sequence context when measuring effects of reproductive aging on the mutation spectrum.

If gBGC were responsible for some of this mutation spectrum divergence, we would expect to observe a systematic difference between species with high effective population size (which experience more gBGC) and species with low effective population size (which experience less gBGC). We did find Watterson's *θ*, an indicator of recent effective population size, to be correlated with the 1-mer spectrum (*r* = 0.55, *P* < 0.005, Bonferroni α = 0.007) and 3-mer mutation spectrum (*r* = 0.48, *P* < 0.003, Bonferroni α = 0.007) (Fig. 3c). These correlations remained significant when using the phylogenetically aware version of the Mantel test (*P* < 0.0008 and *P <* 0.0004, respectively) (supplementary Fig. S14, Supplementary Material online). We note that the correlations between Watterson's *θ* and 1-mer and 3-mer mutation spectra distances are still weaker than the correlations between these mutation spectra and phylogenetic distance (*r* = 0.68, *P* < 8e−6 and *r* = 0.82, *P* < 3e−7, for the 1-mer and 3-mer spectrum, respectively; Fig. 3a and c), further indicating that differences in biased gene conversion strength driven by effective population size are likely not strong enough to fully explain the phylogenetic signal we observe in the 1- and 3-mer mutation spectra. We also find that the correlation of mutation spectrum divergence with Watterson's *θ* disappears once we partition the 3-mer mutation spectrum by 1-mer mutation type, though we showed earlier that the 3-mer subspectrum of each 1-mer type still varies across the phylogeny. This is consistent with our expectation that gBGC acts the same way on all mutations that are part of the same 1-mer mutation class (Fig. 3d, supplementary Fig. S14, Supplementary Material online).

### Evolution of Mutation Rate Dependence on Extended Sequence Context

Although 1-mer and 3-mer mutation spectra are commonly used to study mutational patterns in datasets of modest size, a few studies of human genetic variation have found that mutation rate can depend on extended sequence context over *k*-mers of size 7 or greater (Aggarwala and Voight 2016; Carlson et al. 2018; Liu and Samee 2023; Adams et al. 2023). In theory, more mutational categories could yield greater power to resolve distinct mutagenic processes, but this power can only be realized given sufficient data to fill out rare mutational categories. A study of variation in 5-mer and 7-mer spectra of human populations yielded mixed results, finding some indications that 3-mer sequence context varied more among populations than extended sequence context did (Aikens et al. 2019). A few differences in 5-mer and 7-mer context dependence were observed among human populations, but it is unclear whether these results are robust to quality issues later identified in the low coverage 1,000 Genomes data (Anderson-Trocmé et al. 2020).

**Fig. 4.**
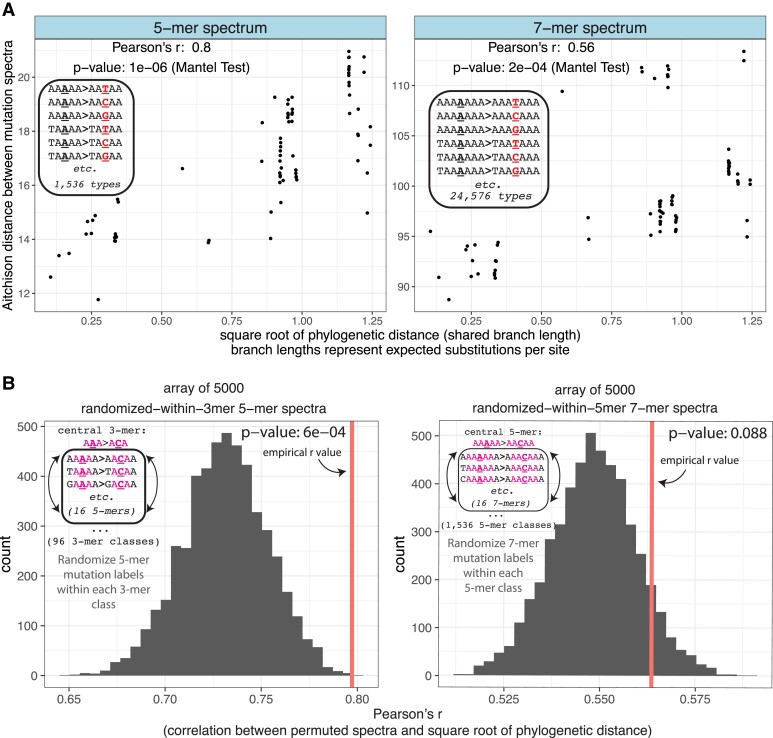
The 5-mer mutation spectrum exhibits additional phylogenetic signal beyond what is inherited from the 3-mer mutation spectrum. a) 5-mer and 7-mer spectra show significant phylogenetic signal. Distances were calculated based on CLR-transformed mutation spectra such that each point represents a single between-species comparison (e.g. human-house mouse). *P*-values were calculated using the Mantel Test with 9,999,999 permutations. A version of these plots with each point labeled is shown in supplementary Fig. S19, Supplementary Material online. b) The empirical 5-mer mutation spectrum is significantly more correlated with the phylogeny than a distribution of 5,000 control spectra generated by randomizing 5-mer mutation counts based on genomic target size within each 3-mer equivalence class (*P* < 6e−4, left panel). In contrast, we observe no significant difference in phylogenetic signal between the empirical 7-mer spectrum and 7-mer spectra that are randomized within 5-mer equivalence classes (*P* > 0.088).

The first 2 principal components of higher-dimensional mutation spectrum variation explain less variance than we observed in PCAs of 1-mer and 3-mer spectra, but clusters within species and higher-order clades appear more visually distinct, with lower cosine similarities between the most distant pairs of species (supplementary Figs. S16 to S18, Supplementary Material online). Higher-dimensional mutation spectra have significant but somewhat weaker phylogenetic signal compared to the 3-mer mutation spectrum (Fig. 4a, see additional 5-mer and 7-mer spectrum and subspectrum phylogenetic signal details in supplementary Note S1 and Figs. S19 to S23, Supplementary Material online).

If a phylogenetic signal exists at the 3-mer level, a Mantel test on the 5-mer mutation spectrum is likely to show a phylogenetic signal even if the effects of extended sequence context on mutation rate are invariant among species. We therefore devised a permutation test to investigate whether each 5-mer's species-specific mutation rate is conditionally independent of the phylogeny after controlling for variation of the 3-mer mutation rate among species. The test involves randomizing the distribution of 5-mer counts within 3-mer equivalence classes to generate 5,000 control spectra per species where the dependence of mutation rate on nonadjacent nucleotides is eliminated (SI Methods). We then compared the empirical correlation of mutation spectrum distance and phylogenetic distance to that of the randomized 5-mer control spectra (Fig. 4b; see supplementary Fig. S24, Supplementary Material online for an example comparing the phylogenetic signal of a single randomized 5-mer replicate to that of the empirical 5-mer spectrum).

We find that the empirical 5-mer spectrum distances are significantly more correlated with phylogeny compared to the 5-mer data that was randomized within each 3-mer class (*P* < 6e−4, 5,000 permutations; Fig. 4b, left panel). A similar analysis indicates that the 7-mer spectrum does *not* contain more phylogenetic signal than the dataset that is randomized to remove information beyond the 5-mer level, at least at our limited sample sizes (*P* < 0.09, 5,000 permutations; Fig. 4b, right panel). After removing the lowest-diversity species in order to sample more SNPs, we see an increased separation between the empirical and permuted 5-mer spectra (*P* < 2e−4) but a decrease in the suggestive difference between the permuted and empirical 7-mer spectra (*P* > 0.75) (supplementary Fig. S25, Supplementary Material online).

As seen for 1-mer and 3-mer spectra, the phylogeny explains 5-mer and 7-mer mutation spectrum divergence consistently better than our list of technical confounders (such as reference genome contiguity) and biological confounders (such as age at first reproduction) (supplementary Fig. S26 and Note S1, Supplementary Material online). The 7-mer spectrum and several of its 1-mer subspectra are significantly correlated with scaffold N50, indicating that this technical confounder may play an important role in shaping spectra at extended sequence contexts (supplementary Note S1; Figs. S27 and S28, Supplementary Material online). However, this dependence on scaffold contiguity may be driven by the vaquita porpoise, which has the highest scaffold contiguity of any species, but also the sparsest 7-mer mutation spectrum due to its extremely low genetic diversity, and clusters apart from all other species in 7-mer spectrum PCA (supplementary Fig. S16, Supplementary Material online). An analysis that excludes the vaquita results in no significant correlation being seen between scaffold N50 and 7-mer mutation spectrum distance after correction for multiple testing (supplementary Note S1 and Fig. S25, Supplementary Material online). These analyses indicate that spectra based on extended sequence context dependencies should be interpreted with caution in modestly sized datasets such as this one due to concerns over data sparsity.

### Variation in Motif Hypermutability and Hypomutability Across Species

**Fig. 5.**
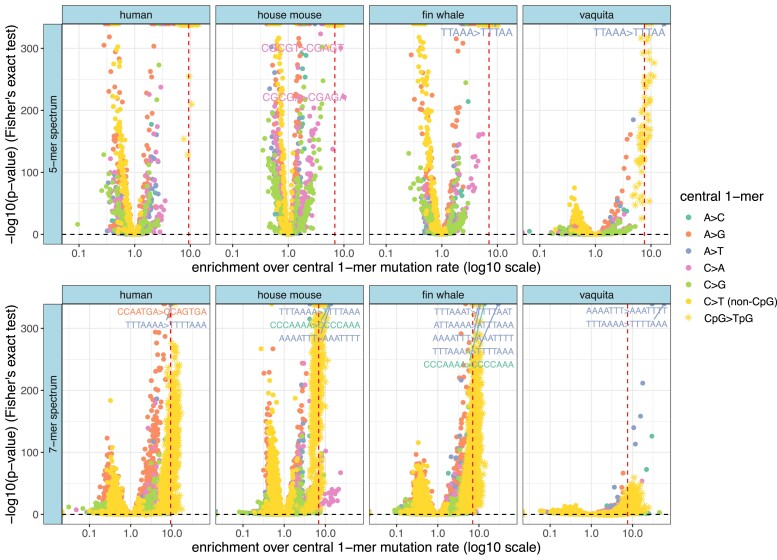
A small subset of 5-mers and 7-mers are hypermutable above the CpG > TpG level. A comparison of the mutabilities of each 5-mer or 7-mer mutation type relative to the background mutation rate of its central 1-mer (e.g. AT**C**CA > AT**T**CA rate divided by C > T rate). The *x*-axis represents the ratio of a particular *k*-mer's rate (counts divided by target size) over the rate of its central 1-mer (counts divided by target size). The *y*-axis is the −log10(*P*-value) from a 2-sided Fisher's exact test. The horizontal black dashed line represents the Bonferroni-corrected statistical significance threshold, and the red vertical dashed line is the species-specific mutability of CpG > TpG dimers relative to the background C > T rate (supplementary Table S3, Supplementary Material online). *k*-mers are colored by central mutation type. Counts are not rescaled to human targets or downsampled since cross-species comparisons are not occurring. Only 4 species are shown: human as a baseline for comparison, house mouse which has a large number of outlying *k*-mers compared to other species, and the 2 cetaceans (fin whale and vaquita porpoise) which show extraordinary enrichment of a particular *k*-mer (TTT**A**AAA > TTT**T**AAA enriched >30-fold above the A > T rate). A small subset of the ∼100 outlying non-CpG > TpG 7-mers are labeled. Supplementary Tables S4 and S5, Supplementary Material online have lists of significantly enriched 5-mers and 7-mers that exceed the CpG > TpG enrichment level. Note that enrichment *P*-values depend on the number of observed SNPs as well as fold enrichment, as evident from the trend toward lower *P*-values in the low-diversity vaquita. See supplementary Figs. S29 to S34, Supplementary Material online for other species’ enrichment profiles and results when CpG islands are included.

A few sequence motifs, such as CpGs, are extremely hypermutable, with mutation rates nearly an order of magnitude above baseline. To measure how the frequency and magnitude of extreme hypermutability varies among species, we used a 2-sided Fisher's exact test to systematically compare *k*-mer-based mutation rates to the average mutation rate of the nested 1-mer (Fig. 5). Unsurprisingly, many *k*-mer mutation rates are significantly different from the nested 1-mer rate after Bonferroni correction, but the 4 N**C**G > N**T**G 3-mers are consistently the most hypermutable 3-mer types across species: enrichment of CpG > TpG above the background C > T rate ranges from 6.6× (orangutan) to 9.3× (human) across the species surveyed (supplementary Table S3 and Fig. S29, Supplementary Material online). If we include mutations occurring in CpG islands, which tend to be hypomethylated and therefore have lower CpG > TpG mutation rates than the rest of the genome (Carlson et al. 2018), CpG > TpG enrichment above C > T background drops to values ranging from 4.8× (orangutan) to 6.7× (polar bear) (supplementary Table S3 and Fig. S30, Supplementary Material online).

As previously seen in humans, certain non-CpG 5-mer and 7-mer motifs actually have mutation rates that are more significantly elevated than those of CpG-containing motifs. This could be due to a myriad of factors, including technical factors such as sequencing chemistry and bioinformatics as well as biological factors such as transposase nicking, mutator activity, transcription factor interference with DNA repair, and exogenous damage. We observed 5 distinct hypermutable non-CpG > TpG 5-mer mutation types (Fig. 5, supplementary Table S4 and Fig. S31, Supplementary Material online): in both cetacean species (fin whale and vaquita porpoise) and the polar bear, TT**A**AA > TT**T**AA is enriched ∼9 to 17× over the A > T rate (*P*-values < 2e−308). The rest of the non-CpG-transition 5-mer hypermutability is observed in mice and consists of C > A mutations in CpG-rich motifs: in *Mus musculus* and *Mus spretus*, CG**C**GT > CG**A**GT (8 to 10×, *P*-values < 1e−200) and CG**C**GA > CG**A**GA (8 to 15×, *P*-values < 1e−148) were significantly enriched above the C > A rate. In *Mus spretus*, CG**C**AA > CG**A**AA (7×, *P*-value < 2e−308) and CG**C**GG > CG**A**GG (8×, *P*-value < 1e−190) were additionally enriched. Note that mice have the highest genetic diversity of any species in our dataset, which should allow for the detection of hypermutable motifs with greater precision and recall than can be achieved with data from less diverse species. If CpG islands are included in the mutation spectrum (shifting the CpG > TpG threshold downward), the TT**A**AA > TT**T**AA enrichment crosses the CpG > TpG threshold in 3 additional species: brown bear, bonobo, and chimpanzee; supplementary Fig. S32 and Table S4, Supplementary Material online).

At the 7-mer level, we observe 101 distinct non-CpG > TpG mutation types that exceed the CpG > TpG fold-enrichment threshold in at least one species (161 if CpG islands are included, lowering the CpG > TpG enrichment threshold) (Fig. 5, supplementary Table S5; Figs. S33 and S34, Supplementary Material online). As seen for 5-mers, mice have the largest number of hypermutable 7-mers, with *Mus spretus* having over 60 types with greater enrichment than CpG > TpG sites, the majority of which are C > A 7-mers. *Mus musculus* had over 40 enriched types (majority C > A).

In humans, Carlson, et al. (2018) previously reported that the only 7-mer more hypermutable than CpG-containing 7-mers was TTT**A**AAA > TTT**T**AAA. Aikens, et al. (2019) also noted that this motif appears to be slightly more hypermutable in Africans compared to Europeans. We find that TTT**A**AAA > TTT**T**AAA is one of the most hypermutable 7-mer types in every species in our study, with enrichments ranging from 9×-59× above species-specific A > T rates (Fig. 5b, supplementary Figs. S33 and S34, Supplementary Material online). Its hypermutability is most extreme (30 to 59× above background) in the fin whale and vaquita sister lineages. We note that the fin whale and vaquita samples were sequenced on different platforms (NovaSeq6000 and HiSeqX, respectively), processed by different researchers, and mapped to reference genomes of very different assembly quality (a highly fragmented assembly was used for the fin whale study, while a highly contiguous chromosomal-level assembly was available for the vaquita). Despite this discrepancy, fin whale and vaquita have similar hyper-enrichments of TTT**A**AAA > TTT**T**AAA, as well as shared enrichments of other repetitive 7-mers.

### Clock-like Catalogue of Somatic Mutations in Cancer Signatures Are Not Phylogenetically Distributed

The underlying mutational processes that generate mutation spectrum patterns can be described as “mutational signatures” of known or unknown etiology. Mutational signatures are frequently used in the cancer literature to link particular environmental exposures or DNA proofreading defects to observed 3-mer somatic mutation spectrum patterns (Alexandrov et al. 2013), and the resulting signatures are maintained in the Catalogue of Somatic Mutations in Cancer (COSMIC) database (Tate et al. 2019). Only 2 COSMIC single base substitution (SBS) signatures are consistently inferred to contribute to germline mutagenesis: SBS1 and SBS5 (supplementary Fig. S35, Supplementary Material online) (Alexandrov et al. 2015; Rahbari et al. 2016; Hamidi et al. 2021; Moore et al. 2021). SBS1 has a known etiology: the deamination of methylated cytosine, resulting in C > T mutations in 3-mers that contain a central CpG sequence. SBS5 has an unknown etiology but is thought to represent a background endogenous mutational process, given its ubiquity and clock-like accumulation pattern.

**Fig. 6.**
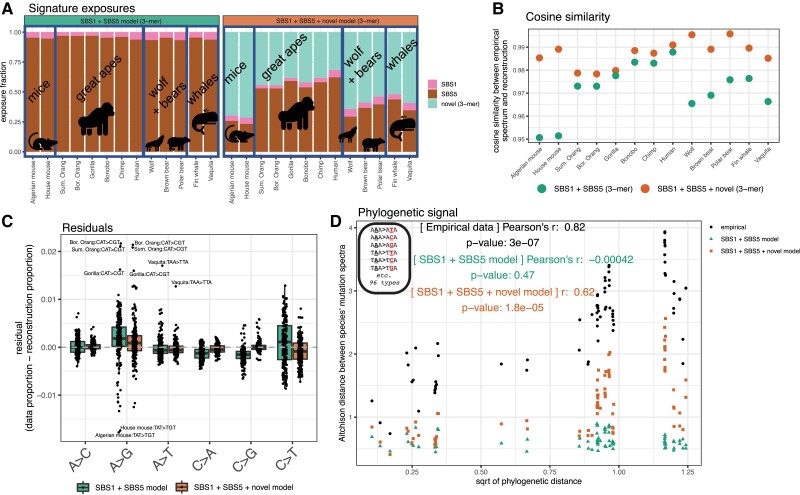
Clock-like COSMIC cancer signatures fail to reconstruct 3-mer spectra. a) Left panel: exposures to COSMIC cancer signatures SBS1 (pink) and SBS5 (brown), derived from human somatic data. Right panel: exposures to a model containing SBS1, SBS5, and an additional third novel signature (teal) extracted from the data. Broad phylogenetic clades are outlined and labeled. Signatures are shown in supplementary Fig. S35, Supplementary Material online. b) Cosine similarity between the empirical spectrum and the spectrum reconstructed using either only SBS1 + SBS5 or the SBS1 + SBS5 + novel signature. Higher cosine similarity indicates a better reconstruction of the data. c) Residuals (observed proportion minus reconstructed proportion) for each spectrum. Boxplots are made up of the 96 3-mer mutation types for all species, separated by their central 1-mer type, with the most extreme outliers labeled. Negative residuals indicate the model reconstruction overestimating a proportion of a mutation type, positive residuals indicate an underestimate. The simple SBS1 + SBS5 model is indicated in green, the SBS1 + SBS5 + novel signature model in orange. d) Result of Mantel test on the correlation between Aitchison distance between species reconstructed 3-mer spectra under each model (green triangles and orange squares) and the square root of phylogenetic distance, overlaid on results based on empirical spectra (black circles). *P*-values based on 9,999,999 permutations.

To determine whether the combination of these 2 signatures could explain the variation in our mammalian 3-mer spectrum data, we used the *R* package *sigfit* (Gori and Baez-Ortega 2020), to model our empirical mutation spectra as linear combinations of “exposures” to SBS1 and SBS5 (Methods). We found that *sigfit* inferred highly similar levels of exposure to SBS1 and SBS5 in each species in our dataset (Fig. 6a, left panel). The corresponding mutation spectrum reconstructions fit the empirical data with high cosine similarity (0.95 to 0.99) (Fig. 6b), albeit with biased residuals (across species, the model consistently underestimates the fractions of A > Gs and C > Ts while overestimating the abundance of C > As and C > Gs) (Fig. 6c).

Despite the high cosine similarity between the empirical mutation spectra and SBS1 + SBS5 reconstructions, we find that the reconstructed mutation spectra have no significant phylogenetic signal (*r* = 0, *P* > 0.4, Mantel test with 9,999,999 permutations; Fig. 6d). Given the strength of the phylogenetic signal in the empirical data, our results indicate that an important source of clade-specific mutation spectrum variation is missing from the SBS1 + SBS5 model.

To investigate what the SBS1 + SBS5 model is failing to capture in the data, we used *sigfit* to infer exposures to SBS1 and SBS5 jointly with an additional novel signature representing some uncharacterized mutational process or processes (signatures shown in supplementary Fig. S35, Supplementary Material online). Although the novel signature inferred by *sigfit* is still fairly similar to SBS5 (cosine similarity 0.94), introducing this signature increased the phylogenetic signal predicted by the model. Exposure to the novel signature is inferred to be highest in mice and lowest in great apes (Fig. 6a, right panel), perhaps reflecting the fact that SBS5 was inferred from human data and is less tailored to the mutational processes of more distantly related species. This 3-signature model still fails to reconstruct the full phylogenetic signal of the empirical 3-mer data (Fig. 6d), indicating that greater complexity is needed to model the cladistic mutation spectrum patterns we observe between mammals’ 3-mer spectra.

### Reproductive Aging Signatures Inferred From Human Data Capture 1-Mer Mutation Spectrum Differences Among Mammalian Species

After seeing that SBS1 and SBS5 cannot explain the phylogenetic signal of the 3-mer mutation spectrum, we turned our attention to a second model of mutation spectrum etiology that attempts to explain differences at the less complex 1-mer mutation spectrum level. A previous human de novo mutation study trained a Poisson regression model to capture the dependence of the mutation spectrum on paternal and maternal age (due to data sparsity, 3-mer mutation spectrum effects were not inferred) (Jónsson et al. 2017), and 2 studies have argued that this reproductive aging model may largely explain 1-mer mutation spectrum differences observed among human populations (Coll Macià et al. 2021; Wang et al. 2023). This human reproductive aging model also appears sufficient to explain de novo mutation spectrum variation within a small pedigree of domestic cats, after appropriate rescaling for differences in lifespan and the timing of puberty (Wang et al. 2022). However, other studies have questioned the ability of a parental age model to explain the full range of mutation spectrum variation even in humans (Gao et al. 2023; Ragsdale and Thornton 2023).

To test whether a reproductive aging model derived from human data is able to explain the variation we observe at the 1-mer level, we attempted to reconstruct our species’ 1-mer mutation spectra using a linear combination of exposures to 3 parental aging signatures derived from mutation patterns observed in human families by Jónsson et al. (2017). Since Jónsson et al. modeled parental mutation contributions to the 1-mer mutation spectrum using a regression, we transformed this regression model into a mutational signature model by translating the slopes into maternal and paternal age signatures and combining the maternal and paternal regression outputs at the age of puberty into a “young parent” signature whose exposure is expected to be highest in the children of young parents (signatures shown in supplementary Fig. S36, Supplementary Material online). As in Wang et al. (2023) and Gao et al. (2023), we excluded CpG > TpG mutations from the signatures and data to avoid confounding by potentially elevated levels of homoplasy and mismapping affecting CpG polymorphisms.

**Fig. 7.**
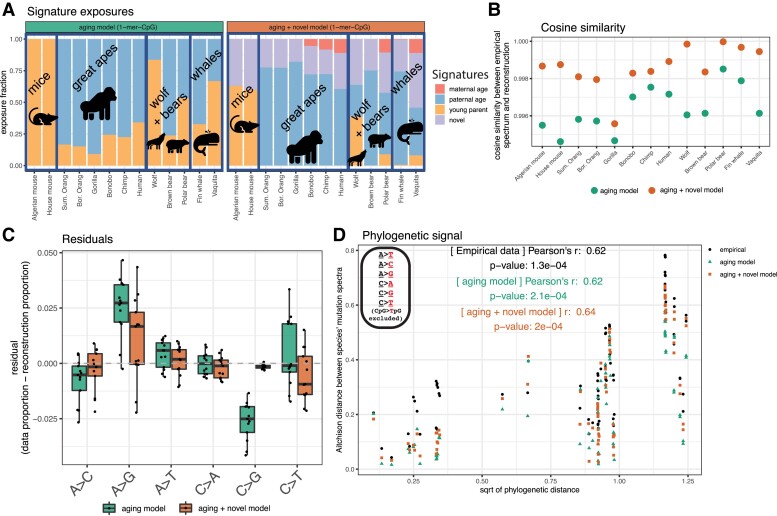
Human reproductive aging signatures can reconstruct the phylogenetic signal of the 1-mer mutation spectrum. a) Left panel: exposures to 3 reproductive aging signatures derived from human trio data in Jónsson et al. (2017) maternal age (red), paternal age (blue), young parents (gold). Right panel: exposures to a model containing an additional fourth novel signature extracted from the data (purple). Broad phylogenetic clades are outlined and labeled. Signatures are shown in supplementary Fig. S36, Supplementary Material online. b) Cosine similarity between the empirical spectrum and the spectrum reconstructed using either the aging signatures alone, or the aging signatures + novel signature. Higher cosine similarity indicates a better reconstruction of the data. c) Residuals (observed proportion—reconstructed proportion) for each spectrum. Boxplots are made up of the 6 1-mer mutation types (CpG > TpG mutations excluded) for all species. Negative residuals indicate the model reconstruction overestimating a proportion of a mutation type, positive residuals indicate an underestimate. The simple 3-signature aging model is indicated in green, the aging + additional novel signature in orange. d) Result of Mantel test measuring correlation between species’ reconstructed 1-mer-minus-CpG spectra under each model (green triangles and orange squares) and the square root of phylogenetic distance, overlaid on results based on empirical spectra (black circles). *P*-values based on 9,999,999 permutations.

We used *sigfit* to infer exposures to these reproductive aging signatures in our 1-mer mammalian mutation spectrum data (Fig. 7a, left panel). The reproductive aging model fits all species’ 1-mer mutation spectra with high cosine similarities (>0.99), but also with biased residuals [across species, the model predicts too many C > Gs and too few A > Gs (Fig. 7b and c)]. This bias may be due in part to the fact that the model was trained on de novo mutation data and then fit to polymorphism data. The residuals do not conform to the expected action of biased gene conversion, as C > G is a GC-conservative mutation type.

Despite these biased residuals, the reproductive aging model is able to capture the phylogenetic signal that is present in the empirical 1-mer mutation spectrum data (Fig. 7D; reproductive aging model *r* = 0.62, *P* < 2.4e−4; empirical 1-mer-minus-CpG spectra *r* = 0.62, *P* < 1.3e−4). We found that adding an additional novel signature extracted from the data (supplementary Fig. S36, Supplementary Material online) improved the fit to the data and reduced the residual bias (Fig. 7b and c), without changing the strength of the phylogenetic signal captured by the reconstructed spectra (Fig. 7d).

### Early Reproduction is Associated With Similar Mutational Biases Across Disparate Clades of Mammals

It is particularly intriguing that the reproductive aging exposures assign most mouse mutations to the young parent signature (Fig. 7a, left panel), possibly because mice reproduce at much younger ages (9 to 11 wk of age) than any of the other species. The second- and third-highest young parent exposures are inferred in wolves and vaquita, which are the second- and third-youngest reproducers in our dataset on average, respectively (∼1 yr and ∼5 yr age at first reproduction). The mice, wolves, and vaquita all have lower Aitchison distances between their 1-mer and 3-mer mutation spectra than comparisons between species with similar or greater phylogenetic distance (supplementary Figs. S4 and S37 , Supplementary Material online), which, given their similar levels of exposure to the young parent signature, may be driven by similarities between these species’ reproductive times relative to other species.

We were able to reproduce this trend using de novo mutation data from mice (Lindsay et al. 2019) and an independently generated wolf polymorphism dataset (Mooney et al. 2023), consistently finding that the species’ 1-mer spectra that were most similar to that of mice were the wolf's and vaquita's 1-mer spectra (supplementary Fig. S37, Supplementary Material online). These results suggest that although the reproductive aging model cannot fully reconstruct these species’ polymorphism spectra in an unbiased way, the reproductive aging signatures are consistent with some of the major 1-mer-level differences that exist between different mammalian clades, which may make species with more similar reproductive strategies have more similar 1-mer spectra.

## Discussion

### Mammalian Polymorphisms Reveal a Hierarchy of Clade-specific Mutational Processes

We have extracted mutation spectra from polymorphism data in 13 species spanning 96 million years of mammalian evolution, standardizing these spectra to remove differences caused by the composition of the accessible reference genome. Our pipeline is designed to facilitate comparison between genomically divergent species that have been sampled and sequenced using different methodologies; we expect that this flexibility will enable expansion of these analyses to more species as new data become available. Although we identified a few correlations between mutation spectrum features and bioinformatic confounding variables, we consistently found phylogenetic distance to be a stronger predictor of mutation spectrum divergence between species. Our results support the hypothesis that the mutation spectrum evolves over time due to slight increases in the rates of some mutation types and slight decreases in the rates of other mutation types.

Previous papers have used principal component analysis to demonstrate that closely related populations appear to have more similar mutation spectra than more distantly related populations (Harris and Pritchard 2017; Dumont 2019; Goldberg and Harris 2022). Here, we utilized the classical phylogenetic Mantel test to quantify the significance of this correlation, a technique that we recently used to quantify mutation spectrum evolution across the phylogeny of severe acute respiratory syndrome coronavirus 2 (SARS-CoV-2) (Bloom et al. 2023). We expect this test to scale well to future datasets containing even more species and populations, and it is adaptable for testing how much variation among mutation spectra is captured by particular mutational signature models or subspectra of interest.

We investigated several nested *k*-mer mutation spectra and found the strongest phylogenetic signal at the 3-mer context level. However, we also found that a small number of 7-mer mutation types exhibit hypermutability that varies conspicuously among species. In particular, the 7-mer TTTAAAA, whose A > T mutation rate is extremely elevated in humans, has a consistently high mutation rate across mammals but an exceptionally high mutation rate within whales. A previous study (Carlson et al. 2018) hypothesized that TTT**A**AAA > TTT**T**AAA hypermutability is caused by LINE-1 transposase activity, which preferentially cuts into specific genomic motifs in a manner that is susceptible to error-prone repair. Since LINE-1 elements have a documented pattern of hyperactivity in Minke whales (Ivancevic et al. 2016), the transposase nicking mechanism might explain the observed interspecies differences in the rate of this outlier mutation type. Although repetitive *k*-mers like TTTAAAA might have an elevated susceptibility to sequencing errors, the hypermutability of this mutation type does not appear to correlate with genome assembly quality or sequencing coverage.

### Power and Limitations of Polymorphism Data for Mutation Spectrum Analysis in Nonmodel Organisms

A limitation of our study is the fact that we inferred mutation spectra from polymorphisms, which typically exhibit systematic differences from de novo mutation spectra ascertained in the same species (Zhu et al. 2014; Carlson et al. 2018; Wang et al. 2022; Ragsdale and Thornton 2023). These polymorphisms are descended from mutations that occurred many generations ago and are impacted by gBGC, which enriches the spectrum for A > G and A > C mutations while depleting it of C > A and C > T. Since the strength of gBGC is proportional to the effective population size, it has potential to create mutation spectrum differences between large, outbred populations (such as mouse populations) and more inbred species such as the vaquita porpoise. However, gBGC is not known to impact A > T or C > G mutations or to influence the 3-mer subspectra of any 1-mer mutation type. In contrast to this expectation, we consistently find phylogenetic signals in subspectra of mutations that each have the same gBGC selective modality (positively selected A > G plus A > C; negatively selected C > A plus C > T; neutrally evolving A > T plus C > G). This finding, combined with the correlation between effective population size and mutation spectrum divergence being weaker than that of phylogenetic distance, suggests that mammalian mutation spectrum variation must be driven by forces other than gBGC. Gao et al. (2023) recently used a similar partitioning strategy to confirm that gBGC cannot be the driver of most signals of human mutation spectrum evolution.

It is possible that these mutation spectra are confounded by selective forces other than gBGC, such as selection to preserve gene regulatory motifs that might be unique to particular lineages. This possibility will be important to investigate as gene regulatory grammar becomes better understood in nonmodel species.

Although polymorphisms are biased by evolutionary processes, they will likely continue to be indispensable for the study of mutation spectrum evolution given that comparably large numbers of de novo mutations are not possible to sample. A sample of 5 unrelated individuals per species allowed us to sample hundreds of thousands of variants and quantify context-dependent mutation spectra with higher precision than would be possible using de novo mutation sets that typically number in the high 10s or low 100s. This precision is essential given that normal germline mutation spectra are much more similar to one another than pathological cancer mutation spectra—all of the 1-mer and 3-mer mutation spectra extracted from species included in this study have pairwise cosine similarities in excess of 0.92. Although these differences are small in magnitude, our Mantel test results show that they exist along a robust hierarchy where more closely related species accumulate mutations in more similar sequence contexts.

### Existing Models of Germline Mutational Signature Etiology Do Not Fully Capture Phylogenetic Signal

Mutational signature deconvolutions of cancer spectra typically assume that a model fits a dataset well if the cosine similarity between the model and the data is greater than 0.95 (Blokzijl et al. 2018; Alexandrov et al. 2020; Gori and Baez-Ortega 2020). Our results indicate that this threshold is likely too permissive for reconstructing germline mutational spectra, at least at the level of precision that is needed to capture the drivers of mammalian mutation spectrum evolution. We can see this by considering the SBS1 + SBS5 clock-like cancer signature model, which captures none of the 3-mer spectrum's phylogenetic signal yet fits each species’ spectrum with cosine similarity greater than 0.95.

Although the SBS1 + SBS5 model and the reproductive aging model achieve similar cosine similarity fits the data, the significant phylogenetic signal captured by the reproductive aging model suggests that it better encapsulates some of the forces that are driving mutation spectrum evolution. It is particularly intriguing that the mouse and the gray wolf have the shortest ages at first reproduction in our dataset and also the lowest exposures to the paternal and maternal aging signatures. However, if the parental age model captured all mutation spectrum variation among mammals, we would expect this model to explain all the variation of the 1-mer spectrum between species with unbiased residuals, which is not the case. This implies that either parental aging cannot explain all mutation spectrum variation between species, or the Jónsson et al. (2017) model does not fully capture the mutational effects of parental aging, perhaps due to the sparsity and bioinformatic complexity of the underlying human mutation data.

Importantly, it is not known how human reproductive aging affects the 3-mer mutation spectrum, as currently available de novo mutation data are too sparse to determine this with precision. It is possible that maternal and paternal aging signatures have different context dependencies that might explain the phylogenetic signal of the 3-mer and 5-mer spectra. In the absence of such data, our results suggest that the reproductive aging model may be an important contributor to mammalian mutation spectrum variation, particularly at the 1-mer level, but that additional factors such as mutator alleles or environmental mutagens may be needed to fully explain the 3-mer phylogenetic signal we observe. The correlation of 3-mer and higher-order mutation spectra with reproductive lifespan and age at first reproduction adds additional evidence that these life history features play a role in shaping mutation spectra. However, since these correlations are weaker than the mutation spectrum's phylogenetic signal, they do not support the idea that life history is the primary driver of mutation spectrum differences between species. The significance of the correlations between these reproductive traits and the mutation spectrum should be interpreted with some caution, as the Mantel test can suffer from an elevated false positive rate when comparing correlations between traits compared to when it is used to measure the phylogenetic signal of a trait (Harmon and Glor 2010; Guillot and Rousset 2013), though the fact that the traits remain significantly correlated with the mutation spectrum in a phylogenetically aware version of the Mantel test increases confidence in the results.

In summary, the observed variation of the mutation spectrum across the phylogeny is consistent with theoretical expectations about a heritable polygenic trait. If germline mutations are generated by several different mutational processes and the rate of each process is subject to weak selective constraint, then as species evolve, each of their mutation spectra should perform a random walk through a multidimensional space. Other explanations of the data are also possible: to the extent that closely related species tend to inhabit similar environments and reproduce via similar strategies, environmental mutagens, and conserved reproductive aging signatures might create phylogenetic signal that is consistent with our expectations of a mutator allele, particularly for the lower-dimensional 1-mer spectrum. To disentangle these possibilities, it will be necessary to sample mutation data from a wider variety of species that independently came to inhabit similar environments or reproductive niches, as well as species that recently came to inhabit new environments (as humans did). We anticipate that such mutation data will become increasingly available over the coming years and that the methodology presented in this paper will allow for nuanced modeling of the mutagenic effects of genotype versus environment.

## Methods

*Note:* extensive additional methodological details are presented in the SI Methods.

### Polarization

To assign mutations to spectrum types (e.g. T**A**C > T**G**C), the ancestral allele state must first be determined. Ancestral allele assignments were previously generated for the human genome as part of the 1,000 Genomes Project (Auton et al. 2015) and for the nonhuman great ape species by Goldberg and Harris (2022). We assigned ancestral states probabilistically using *est-sfs* (Keightley and Jackson 2018) using outgroup species sequences and allele frequencies for the remaining species in our dataset. Details on polarization approaches are in SI Methods.

### Generating Mutation Spectra From Polymorphism Data

The mutation spectrum represents the distribution of relative abundances of different mutation types, sometimes calculated from all derived alleles present in one individual genome and sometimes calculated from all sites that are variable within a larger population sample. In the simplest form of the mutation spectrum, which we call the 1-mer spectrum, mutations are classified into 6 types (A > T, A > C, A > G, C > T, C > G, C > A) with DNA strand complements collapsed. This can be expanded into a 7-dimensional “1-mer + CpG spectrum” by separating C > T mutation types into those that are found in a CpG context and those that are not. CpG > TpG mutations can also be removed from the spectrum entirely (“1-mer-minus-CpG spectrum”).

Finer-grained mutation spectra can be computed by subdividing these basic mutation types by their flanking sequence context. In the popular 3-mer spectrum, mutations are classified by their immediate 5′ and 3′ flanking basepairs, yielding 96 mutation types once reverse complements are collapsed (e.g. T**C**C > T**G**T, A**A**C > A**T**C, A**C**G > A**T**G, etc.). Higher-dimensional spectra are possible as well, including 5-mers (2 bp on either side of the mutating base, e.g. TA**C**CT > TA**T**CT, resulting in 1,536 mutation types), or 7-mers (3 bp on either side of the mutating base, e.g. TTA**C**CTA > TTA**T**CTA, resulting in 24,576 mutation types). These higher-dimensional spectra have considerably more mutation types, which can perhaps aid in the detection of more subtle mutation signatures, but can also lead to issues of data sparsity.

For a set of 5 individuals randomly sampled from each species in the dataset, we estimated a nested series of spectra at the 1-mer, 1-mer + CpG, 1-mer-minus-CpG, 3-mer, 5-mer, and 7-mer levels from our polymorphism data using the program *mutyper* (DeWitt et al. 2023). Shared variation among individuals was randomly assigned to the spectrum of a single individual so that the same mutation does not contribute to multiple spectra. Spectra at the overall species level were then estimated (the equivalent of summing the per-individual spectra). Details on mutation spectrum generation in SI Methods.

### Correcting for Genome Content and Amount of Genetic Diversity

To compare mutation spectra between individuals and species, the mutation spectra must be corrected for several factors, including genomic content and genetic diversity. The 7-mer, 5-mer, 3-mer, and 1-mer spectra were corrected in the same manner, as described below.

Genomic *k*-mer content may differ across species. For example, one species may have more “ACC” 3-mers in its genome, causing more A**C**C > A**N**C mutations to accumulate due to a larger ACC target size rather than a higher A**C**C > A**N**C mutation rate). To correct for differences in genomic content between humans and any other species (here denoted “species A”), we transformed species A's SNP count $x_{m\to j,A}$ of 7-mer mutation type m→j (*k*-mer *m* mutates to *k*-mer *j*) into a rescaled SNP count $x_{m\to j,A}^{\left(r\right)}$ that is what we would expect to observe if the mutation rate were unchanged but the fraction of the ancestral *k*-mer of mutation type *m* in species A was changed to match the human reference genome:


$$x_{m\to j,A}^{\left(r\right)}\;=\;x_{m\to j,A}\;*\;\frac{\frac{t_{m,h}}{T_{h}}}{\frac{t_{m,A}}{T_{A}}}$$

where *t_m,A_* is the number of times the ancestral *k*-mer of mutation type *m* is observed in species A's reference genome, *T_A_* is the total target count (sum of all *k*-mer targets) in species A's reference genome, *t_m,h_* is the number of times the ancestral *k*-mer of mutation type *m* is observed in the human reference genome, and *T_h_* is the total target count (sum of all *k*-mer targets) in the human reference genome. We rescale all mutation spectra to reflect the human reference genome's *k*-mer composition. Although the choice of human as the standard reference is arbitrary, supplementary Note S2, Supplementary Material online shows that CLR-transformed mutation spectrum comparisons actually do not depend on which genome composition the spectra are rescaled to as long as all spectra are rescaled to reflect the same reference composition.

After rescaling all counts to the same genomic content multinomial downsampling is carried out using the *rmultinom* function in *R* to sample the number of SNPs passing all filters and masks found in the lowest diversity species using the fraction of each mutation type as the multinomial probabilities. The vaquita had the lowest diversity, and so all species were downsampled to match the ∼130,000 SNPs observed in the vaquita dataset. At these lower numbers of SNPs, some mutation categories have mutation counts of 0 (particularly in the high-dimensional 7-mer spectrum, and to a lesser extent, the 5-mer spectrum). Since the CLR transformation is incompatible with mutation counts of zero, we regularized our data by adding a pseudocount of 1 to the number of mutations observed within each type category in each species.

### Reproductive Aging Mutational Signatures

We extracted mutational signatures related to human reproductive aging from Poisson regressions carried out by Jónsson et al. (2017) from Icelandic human trio data, which measured the effects of maternal and paternal age on de novo mutation counts for each 1-mer + CpG mutation class (C > A, C > G, C > T, CpG > TpG, T > A, T > C, T > G in Jónsson et al.). As in Wang et al. (2023) and Gao et al. (2023), we excluded CpG > TpG sites since they are so depleted from variation data compared to de novo mutation data due to purifying selection.

For each mutation class *c*, Jónsson et al. calculate maternal and paternal age slopes $m_{c,mat}$ and $m_{c,pat}$ as well as a y-intercept values $b_{c,mat}$ and $b_{c,pat}$ such that a child who is conceived when their mother's and father's ages are $a_{mat}$ and $a_{pat}$ is expected to inherit $y_{c,mat}$ and $y_{c,pat}$ mutations of type *c* from their mother and father, respectively:


$$y_{c,mat}(a_{mat})=m_{c,mat}\;*\;a_{mat}+b_{c,mat}$$

$$y_{c,pat}(a_{pat})=m_{c,pat}\;*\;a_{pat}+b_{c,pat}$$

We took Jónsson, et al.'s reported values of $m_{c,mat}$, $b_{c,mat}$, $m_{c,pat}$, and $b_{c,pat}$ (located in supplementary Table S9, Supplementary Material online in Jónsson et al.) and transformed these data into 3 mutational signatures. Letting *c'* range over the vector of 1-mer mutation types, the paternal age signature is a 6-dimensional vector with entries $\frac{m_{c,pat}}{\Sigma_{c^{\prime }}\;m_{c^{\prime },pat}}$ (describing the fraction of paternal non-CpG mutations composed of each 1-mer mutation type) and the maternal age signature is the vector $\frac{m_{c,mat}}{\Sigma_{c^{\prime }}\;m_{c^{\prime },mat}}$. Finally, we generated a “young parent” signature that is representative of the mutations occurring in the offspring of 2 parents who reproduce directly after puberty. Assuming puberty occurs at age 13 in humans, we calculate that the entries of this young parent mutational signature vector should be


$$\frac{y_{c,mat}(13)+\;y_{c,pat}(13)}{\Sigma_{c^{\prime }}y_{c^{\prime },mat}(13)+\;y_{c^{\prime },pat}(13)}$$

#### Using *Sigfit* to Extract Mutational Signature Exposures

We used the R package *sigfit* (Gori and Baez-Ortega 2020) to model our germline mutation spectrum data as linear combinations of prespecified mutational signatures. The outputs of *sigfit* are “signature exposures” that specify the proportion of mutations in each species that were generated by each input mutational signature. These exposure proportions are estimated by maximizing the cosine similarity between each species’ mutation spectrum and an exposure-weighted average of all input mutational signatures. Since COSMIC signatures are defined relative to human genome composition, we rescaled all species’ mutation counts to human genome content.

We fit each prespecified signature model to our data using the multinomial model with 10,000 iterations. This procedure was used to fit the SBS1 + SBS5 signatures to the 3-mer spectrum and to fit the reproductive aging model to the 1-mer-minus-CpG spectrum. We then added a novel mutational signature to each of these models using the *fit_extract_signatures* function (multinomial model; 10,000 iterations; adapt_delta = 0.99).

After fitting each of these 4 models, we calculated cosine similarities between each species’ mean reconstructed mutation spectrum (based on mutation signatures and exposures inferred by *sigfit*) and the empirical spectrum that was used to fit the model. We calculated per-species permutation-type residuals by subtracting the reconstructed mutation fraction from the empirical fraction.

To measure whether the reconstructions accurately capture phylogenetic signals, we carried out Mantel tests (9,999,999 permutations) to measure the correlation between species’ reconstructed spectra and the square root of their phylogenetic distance. We then compared these results to Mantel test results of the correlation between the empirical 1-mer-minus-CpG and 3-mer spectra and phylogenetic distance.

## Supplementary material

Supplementary material is available at *Molecular Biology and Evolution* online.

## Supplementary Material

msad213_Supplementary_Data

## Data Availability

Code used to generate spectra and analyses is available on https://github.com/harrispopgen/mammal_mutation_spectra. Data files, including mutation spectra with different transformations, input vcf files, genome masking bed files, ancestral fasta files, mutyper pipeline output files, full results of Fisher's exact test for enrichment/depletion of *k*-mers, and reproductive aging mutation signatures have been placed on Dryad (https://doi.org/10.5068/D1339F) with an extensive readme file describing each set of files.
