# Supplementary material for: Evolution of the Mutation Spectrum Across a Mammalian Phylogeny
Source: Mol Biol Evol. 2023 Sep 28;40(10):msad213. doi: 10.1093/molbev/msad213 (PMC10566577; doi:10.1093/molbev/msad213)
Supplement: msad213_Supplementary_Data [file msad213_supplementary_data.zip › Beichman_SI_Notes_and_SI_Methods.Revision.UpdatedReferences.pdf]

Supplemental Notes

Note S1. Additional information about 5-mer and 7-mer spectrum results.

As in our analyses of the phylogenetic signal of 1-mer and 3-mer spectra, we obtained qualitatively consistent phylogenetic signal results for 5-mer and 7-mer spectra (**Figure 4**) when using an ultrametric tree (**Figure S20**), stratifying spectra by biased gene conversion categories (**Figure S21**), using cosine distance (1- cosine similarity) instead of Aitchison distance to measure distance between spectra (**Figure 22**), and folding the spectrum to eliminate sensitivity to incorrect ancestral allele inference (**Figure S23**).

K_{mult} test. Values of K_{mult} for 5-mer and 7-mer spectra are significant, but considerably lower than those of 1-mer and 3-mer spectra (5-mer: $K_{mult} = 0.1$, $p < 0.001$; 7-mer: $K_{mult} = 0.07$, $p < 0.001$; 999 permutations), which may be due to weaker phylogenetic signal, or indicate that the dimensionality of the mutation spectrum is growing much faster than the number of distinct mutational signatures that make up these mutation spectra (Adams and Collyer 2019) (**Figure S11**).

Sub-spectra, biological and technical confounders. Differences in genetic diversity and age at first reproduction are significantly correlated with 5-mer spectrum distances (**Figure S26**). The phylogeny is also more correlated with 7-mer spectrum distances than any other confounder, but reference genome scaffold N50 is also significantly correlated with 7-mer spectrum distances (**Figure S26**). When 5-mer or 7-mer spectra are separated into sub-spectra based on central 1-mer mutation type (e.g. 256 5-mers that have A>T as the central 1-mer mutation), most sub-spectra continue to show a significant phylogenetic signal, though A>T 5-mers and A>T, A>C and C>T 7-mers do not have significant phylogenetic signal after correcting for multiple testing (**Figure S27-S28**). Reference genome scaffold N50 is more correlated than phylogenetic distance for A>C, A>T, C>A and C>T 7-mer sub-spectra (**Figure S27-S28**).

This dependence on scaffold N50 may be partially driven by the vaquita, which has the highest genome scaffold contiguity of any species (**Table 1**), but also the sparsest 7-mer mutation spectrum due to its extremely low diversity, making it have elevated 7-mer mutation spectrum distance from all other species (an outlier on PC1 in the 7-mer based PCA (**Figure S16B**) and the elevated points in the scatter plot in **Figure 4A**, right panel). When low-diversity species, including the vaquita, are excluded from the analysis as described below, scaffold N50 is *no longer* significantly correlated with the 7-mer spectrum or any of its sub-spectra, though the 7-mer spectrum remains correlated with genetic diversity and age at first reproduction (**Figure S25**).

Data sparsity. The decrease in phylogenetic signal with increased spectrum dimensionality may be due in part to data sparsity. We were able to somewhat mitigate this data sparsity issue by excluding two low-diversity species (vaquita and polar bear) and substituting the higher-diversity Eastern North Pacific fin whale population for the low diversity Gulf of California population. The elimination of these species results in downsampling to ~890k SNPs across species instead of ~130k (**Figure S25**; 5-mer spectrum $r = 0.86$, $p < 2.8e-5$; 7-mer spectrum $r = 0.74$, $p < 3.7e-5$), which increases the amount of phylogenetic signal we measure when looking at the 7-mer spectrum, but still does not yield a better fit than the 7-mer spectrum permuted across 5-mer categories ($p > 0.75$) (**Figure S25B**).

Note S2. Proof that mutation counts and relative rates yield identical estimates of CLR mutation spectrum distances.

The mutation spectrum is sometimes computed using raw mutation type counts, but it is sometimes computed using SNP counts that have been rescaled by target size (i.e. the number of A**A**A>A**C**A SNPs in the genome divided by the number of AAA 3-mers where a SNP might be called if one exists). We refer to these rescaled SNP counts as relative rates since in the absence of natural selection, they should be proportional to the rates at which different mutation types arise. When using relative rates, we have found that Euclidean distances between mutation spectra are largely determined by the abundance of high-rate CpG mutations, while low-rate mutation types, which proportionally may differ greatly between species, have negligible impact (**Figure S38**). Intuitively, mutation spectra that are computed using relative rates look slightly "spikier" than spectra computed from raw counts, with larger contributions due to hypermutable mutation types such as CpGs. In contrast, we have found that CLR-transformed mutation spectrum distance depends more strongly on differences in the abundance on rarer mutation types, and we can show that the value of this distance does not actually depend on whether SNP counts are normalized for genome content or not, as long as all species have been corrected to have the same genome content.

To prove this, we represent the human-genome-content rescaled mutation count of a *k*-mer m mutating to *k*-mer j in species A as ${x^{(r)}}_{m\to j,A}$ (r designates that the count has been rescaled by human genome target content, as in the **Methods**).

A full vector of all rescaled mutation counts for species A is $x_A^{(r)}$.

The target size of *k*-mer m in the human genome (which all species' counts have been rescaled relative to) is $t_{m,h}$, and the vector of target sizes for all mutation types is t_h (note that since each *k*-mer can mutate to three possible mutation types, the length of the vector t_h is three times longer than the vector just of targets themselves, with each target repeated 3x).

The Euclidean distance between the raw SNP vectors $x_A^{(r)}$ and $x_B^{(r)}$ will generally be different from the Euclidean distance between the normalized vectors $x_A^{(r)}/t_h$ and $x_B^{(r)}/t_h$.

However, we will show that the Aitchison distances between these two different ways of scaling the mutation spectra are identical, provided we have rescaled our mutation counts to account for any differences between the target sizes of the genomes from which the two different mutation spectra were sampled (**Figure S39**) (in our study, all counts are rescaled to reflect human reference genome composition; see **Methods**).

CLR transformation involves dividing a value by the geometric mean of the full compositional vector, and taking the natural log:

$$CLR_I = ln\,(\frac{x_I}{GM(x)})$$

where x_I is a value of a compositional vector, and x is the full compositional vector, and *GM* is the geometric mean function.

The geometric mean (GM) has a very useful property:

$$GM\left(\frac{X}{Y}\right) = \frac{GM(X)}{GM(Y)}$$

The Aitchison distance between CLR-transformed vectors x_A and x_B is defined as:

$$d(x_A, x_B) = \sqrt{\sum_{i=1}^{S}\left[ln\left(\frac{x_{i,A}}{GM(x_A)}\right) - ln\left(\frac{x_{i,B}}{GM(x_B)}\right)\right]^2}$$

where S is the length of each vector, $x_{i,A}$ is the ith value of vector x_A and $x_{i,B}$ is the ith value of vector x_B.

Due to the logarithmic property that ln(X)-ln(Y) = ln(X/Y), this equation can also be written as:

$$d(x_A, x_B) = \sqrt{\sum_{i=1}^{S}\left[ln\left(\frac{\frac{x_{i,A}}{GM(x_A)}}{\frac{x_{i,B}}{GM(x_B)}}\right)\right]^2}$$

In the above equation, we can define the mutation spectrum vectors x_A and x_B to either be unnormalized count vectors $x_A^{(r)}$ or to be normalized vectors of the form $x_A^{(r)}\ /t_h$.

If we use the unnormalized mutation count vector $x_A^{(r)}$ to calculate Aitchison distance, then the CLR value for the count (denoted CLR_C for 'count') of mutation type $m \rightarrow j$ in species A is:

$$CLR_{C,m\rightarrow j,A} = ln\left(\frac{x^{(r)}{}_{m\rightarrow j,A}}{GM(x_A^{(r)})}\right)$$

However, if we use the target-normalized mutation count vector

$$x_A^{(r)}\ /t_h\ ,$$

the CLR value for the mutation count for mutation type $m \rightarrow j$ divided by the target size of *m* (denoted CLR_R for 'rate') in species A is:

$$CLR_{R,m\rightarrow j,A} = ln\left(\frac{\frac{x^{(r)}{}_{m\rightarrow j,A}}{t_{m,h}}}{GM\left(\frac{x_A^{(r)}}{t_h}\right)}\right)$$

We can then separate out the numerator and denominator of the geometric mean to obtain:

$$CLR_{R,m\rightarrow j,A} = ln\left(\frac{\frac{x^{(r)}{}_{m\rightarrow j,A}}{t_{m,h}}}{\frac{GM\left(x_A^{(r)}\right)}{GM(t_h)}}\right)$$

Some algebraic rearrangement yields:

$$CLR_{R,m\rightarrow j,A} = ln\left(\frac{x^{(r)}{}_{m\rightarrow j,A}\ *\ GM(t_h)}{t_{m,h} * GM\left(x_A^{(r)}\right)}\right)$$

Similarly, for species B:

$$CLR_{R,m\rightarrow j,B} = ln\left(\frac{x^{(r)}{}_{m\rightarrow j,B}\ *\ GM(t_h)}{t_{m,h} * GM\left(x_B^{(r)}\right)}\right)$$

Note that the target sizes $t_{m,h}$ are identical for species A and B, because both species have had their spectra rescaled to match the human genomic target content.

To calculate the Aitchison distance, we compute the difference between the two CLR values and observe that the target sizes cancel out as follows:

$$ln\left(\frac{x^{(r)}{}_{m\to j,A} * GM(t_h)}{t_{m,h} * GM\left(x_A^{(r)}\right)}\right) - ln\left(\frac{x^{(r)}{}_{m\to j,B} * GM(t_h)}{t_{m,h} * GM\left(x_B^{(r)}\right)}\right) = ln\left(\frac{\frac{x^{(r)}{}_{m\to j,A} * GM(t_h)}{t_{m,h} * GM\left(x_A^{(r)}\right)}}{\frac{x^{(r)}{}_{m\to j,B} * GM(t_h)}{t_{m,h} * GM\left(x_B^{(r)}\right)}}\right)$$

This simplifies to:

$$ln\left(\frac{x^{(r)}{}_{m\to j,A} * \cancel{GM(t_h)} * \cancel{t_{m,h}} * GM\left(x_B^{(r)}\right)}{\cancel{t_{m,h}} * GM\left(x_A^{(r)}\right) * x^{(r)}{}_{m\to j,B} * \cancel{GM(t_h)}}\right)$$

All information regarding genomic target size cancels out, leaving:

$$ln\left(\frac{x^{(r)}{}_{m\to j,A}}{GM\left(x_A^{(r)}\right)} * \frac{GM\left(x_B^{(r)}\right)}{x^{(r)}{}_{m\to j,B}}\right) = ln\left(\frac{\frac{x^{(r)}{}_{m\to j,A}}{GM\left(x_A^{(r)}\right)}}{\frac{\vec{x}_{m\to j,B}}{GM(\vec{x}_B)}}\right) = ln\left(\frac{x^{(r)}{}_{m\to j,A}}{GM\left(x_A^{(r)}\right)}\right) - ln\left(\frac{x^{(r)}{}_{m\to j,B}}{GM\left(x_B^{(r)}\right)}\right)$$

This is simply the difference between the count-based CLR values for species A and B.

A similar argument proves that a CLR distance computed from raw mutation counts is identical to the CLR distance computed from mutation type proportions (**Figure S39**). We reiterate that proportions should only be used as long as target sizes have been rescaled to the same genomic content – otherwise proportions, rates, or counts will reflect species-specific differences in genome content.

Supplemental Methods

Data acquisition

Humans. Byrska-Bishop et al. (2022) sequenced 2,504 human genomes to 30x coverage (Illumina NovaSeq 6000). They mapped these data to the human genome assembly GRCh38 and called genotypes using GATK HaplotypeCaller, with variant quality score recalibration (VQSR) carried out as described in their paper. We downloaded the resulting genotype dataset in *.vcf* format ("1000 Genomes 30x on GRCh38" dataset) from the 1000 Genomes FTP site (https://www.internationalgenome.org/data-portal/data-collection/30x-grch38). We used superpopulation assignments for downstream analyses with the following sample sizes: African (AFR; n = 661), European (EUR; n = 503), South Asian (SAS; n = 489), East Asian (EAS; n = 504), Admixed Americans (AMR; n = 347).

Non-human great apes. Prado-Martinez et al. (2013) carried out whole genome sequencing to mean 25x coverage (Illumina HiSeq 2000) of 79 great ape individuals from the species chimpanzee (*Pan troglodytes*), bonobo *(Pan paniscus*), gorilla (*Gorilla gorilla* and *Gorilla beringei*), Sumatran orangutan (*Pongo abelii*), and Bornean orangutan (*Pongo pygmaeus*). They mapped the short-read sequencing data to the human genome hg18 (NCBI Build 36) and called genotypes using *GATK UnifiedGenotyper* with the following filter criteria: DP < (mean_read_depth/8.0) || DP > (mean_read_depth*3), QUAL < 33, FS > 26.0, MQ < 25, MQ0 ≥ 4 && ((MQ0 / (1.0 * DP)) > 0.1), and sites within 5bp of an indel (Prado-Martinez et al. 2013). We downloaded their processed genotype *.vcf* files from the Great Ape Genome Project (GAGP) (https://www.biologiaevolutiva.org/greatape/). For chimp, one individual (Pan_troglodytes_ellioti-Banyo) was excluded from downstream analyses as it was listed as low quality in the GAGP documentation. For the Western Lowland gorilla (*Gorilla gorilla gorilla*), two individuals listed in the GAGP documentation as being low quality were removed (Gorilla_gorilla_gorilla-X00108_Abe and Gorilla_gorilla_gorilla-KB7973_Porta). Additionally, individuals from the *Gorilla beringei graueri* and *Gorilla gorilla dielhi* subspecies were excluded to reduce population structure in the dataset (Gorilla_beringei_graueri-9732_Mkubwa, Gorilla_beringei_graueri-A929_Kaisi, Gorilla_beringei_graueri-Victoria, Gorilla_gorilla_dielhi-B646_Nyango).

After exclusion of these individuals, the resulting samples sizes were chimpanzee ($n = 24$), bonobo ($n = 13$), gorilla ($n = 25$), Sumatran orangutan ($n = 5$), and Bornean orangutan ($n = 5$).

Wild mice. Harr et al. (2016) sequenced mouse whole genomes to 11-26x coverage (Illumina HiSeq 2000) from natural populations of house mouse (*Mus musculus*), including the subspecies western house mouse (*M. m. domesticus*) and eastern house mouse (*M. m. musculus*), and the Algerian mouse (*Mus spretus*). They also included publicly available data from the southeast-Asian house mouse (*M. m. castaneus*) in their genotype calling pipeline. They mapped reads to the mm10 mouse reference genome and called genotypes using *GATK* best practices, including VQSR. Sample sizes for each species/subspecies are *M. m. domesticus* (Mmd; $n = 27$), *M. m. musculus* (Mmm; $n = 22$); *M. m. castaneus* (Mmc; $n = 10$), *M. spretus* (Ms; $n = 8$).

Gray wolf. The Broad Institute processed whole genome sequences of 676 canids (10-94x coverage) together from a combination of publicly available sequences and newly sequenced individuals (Morrill et al. 2022). The reads were mapped to the dog reference genome (canfam3) and genotypes were called using *GATK HaplotypeCaller*, with hard filters QD < 2.0, FS > 60.0, MQ < 40.0, MQRankSum < -12.5, ReadPosRankSum < -8.0, and GQ < 14.0. We selected a subset of gray wolves (*Canis lupus*) from the dataset and one coyote (*Canis latrans*) to act as an outgroup for polarization (wolves: Wolf03, Wolf08, Wolf19, Wolf20, Wolf24, Wolf27, Wolf29, Wolf31, Wolf32, Wolf33, Wolf34, Wolf35, Wolf36, WO001_895, WO002_732, WO003_636, Wolf40, Wolf41, Wolf42, and coyote: cal_coy). The resulting sample size was gray wolf ($n = 19$).

Fin whale. Nigenda-Morales et al. (2023) sequenced 50 fin whale (*Balaenoptera physalus*) whole genomes (27x coverage; Illumina HiSeqX and NovaSeq6000) from two populations, the Eastern North Pacific (ENP) and the Gulf of California (GOC). They mapped reads to the outgroup minke whale genome (BalAcu1.0) and called genotypes using *GATK HaplotypeCaller*. Additionally, they mapped one humpback whale (*Megaptera novaeangliae*) and one blue whale (*Balaenoptera musculus*) individual to the minke whale genome for use in polarization. Variants were filtered using hard filters, with minimum genotype depth of 8, and maximum as 250% of the mean per-individual depth. Genotypes had to have a minimum Phred score of 20 and have 0.2 ≤ allelic balance ≤ 0.8 for heterozygous genotypes, allelic balance ≥ 0.9 for homozygous reference genotypes, and allelic balance ≤ 0.1 for homozygous alternate genotypes. They also filtered sites based on the *GATK* recommended hard filters (QD < 2.0, FS > 60.0, MQ < 40.0, MQRankSum < -12.5, ReadPosRankSum < -8.0, SOR > 3.0, and QUAL < 30). Sites where >75% of individuals were heterozygous or >20% of individuals contained missing genotypes were also excluded. They excluded six individuals that had low genotype quality, high degrees of admixture, or high kinship to other individuals from the dataset (ENPOR12, ENPCA01, ENPCA09, GOC010, GOC080, GOC111). We excluded one additional low-coverage individual (ENPAK28) from downstream analyses. The resulting sample sizes were fin whales from the Gulf of California (GOC; n = 17) and fin whales from the Eastern North Pacific (ENP; n = 26).

Vaquita porpoise. Robinson et al. (2022) sequenced 20 vaquita porpoise (*Phocoena sinus*) whole genomes from the Gulf of California to 60x coverage (Illumina HiSeqX). They mapped reads to the vaquita reference genome (mPhoSin1.pri, GCF_008692025.1) and called genotypes using *GATK HaplotypeCaller*. They filtered genotypes using the hard filters: QD < 4, FS > 60, MQ < 40, MQRankSum < -12.5, ReadPosRankSum < -8, SOR > 3. Sites at which >75% of individuals were heterozygous were excluded. Heterozygous sites with allele balance < 0.2 or > 0.8 were excluded, as were homozygous genotypes with allele balance > 0.9 or < 0.1. They filtered genotypes with depth less than 1/3 of the mean read depth or greater than 2x mean read depth of the individual. After we excluded five relatives of individuals left in the dataset (z0001663, z0004380, z0004393, z0004394, z0185383), the resulting sample size was vaquita (n = 15).

For all datasets, the genotype data were restricted to biallelic SNPs with the "PASS" designation, indicating that they have passed all filters.

Polar bear and brown bear genotype calling

Since a unified dataset for polar bear (*Ursus maritimus*) and brown bear (*Ursus arctos*) was not available, we carried out genotype calling ourselves. Publicly available fastq files for polar bear and brown bear individuals (**Table S2**) were downloaded from the European Nucleotide Archive (ENA) (Miller et al. 2012; Cahill et al. 2013; Liu et al. 2014; Benazzo et al. 2017; Barlow et al. 2018). Additionally, one black bear (*Ursus americanus*) individual was included for use in polarization (see below) (**Table S2**) (Srivastava et al. 2019). Paired-end reads were mapped to the brown bear (Acc. # GCA_003584765.1) and polar bear (Acc. # GCA_000687225.1) genomes using *paleomix* (Schubert et al. 2014), which removes adapters and PCR duplicates and filters out reads with quality < 30. Variants were called for all scaffolds ≥ 1Mb, corresponding to 2.218Gb of sequence in the polar bear reference genome (out of a total genome size of 2.301 Gb) and 2.253Gb in the brown bear genome (out of 2.328Gb). Note that reads were mapped to the set of all scaffolds, but variants were only called for those ≥ 1Mb using *GATK* (v. 3.7) *HaplotypeCaller*, with all sites emitted and sites with mapQ < 20 filtered out (Van der Auwera et al. 2013). Sites were then joint-genotyped across all individuals using *GATK GenotypeGVCFs*, emitting all sites (variant and invariant).

GATK hard filters were then used to filter sites, excluding variants that had: QD < 2.0, FS > 60.0, SOR > 3.0, MQ < 40.0. MQRankSum < -12.5, ReadPosRankSum < -8.0. Additionally, at the genotype level to be retained the individual genotype had to have GQ ≥ 20, genotype depth that is ≥ 8 and ≤ 250% of the mean depth of the individual, have 0.2 ≤ allelic balance ≤ 0.8 for heterozygous genotypes, allelic

balance > 0.9 for homozygous reference genotypes, and allelic balance < 0.1 for homozygous alternate genotypes. Finally, sites at which > 75% of individuals were heterozygous were filtered out. After filtering, thirteen lower-coverage individuals had < 19.5Gb of sites passing filters, and so were excluded from downstream analyses (006_UARC_SW_BGI_brownbear_20105373,015_UARC_GEO_Ge 017_UARC_RUS_S235,020_UARC_AK_Uarc_EP040,021_UARC_ABC_Uarc_EP050 040_UMAR_SVL_PB1_N23531,041_UMAR_SVL_PB10_N23997,042_UMAR_SVL_PB2_N23604,043_UMAR_SVL_PB3_N23719,044_UMAR_SVL_PB4_N23917,045_UMAR_SVL_PB6_N26028,047_UMAR_SVL_PB8_N7968,048_UMAR_SVL_PB9_N23985).

Brown bears were classified into two geographic groups–the Alaskan Admiralty, Baranof, and Chicagof (ABC) islands and Europe. Two individuals from Alaska or Montana were excluded as outliers originating from outside of these two main populations. After exclusion of these individuals, the resulting sample sizes were polar bear (n = 18) and brown bear from ABC Islands (n = 8) and brown bears from Europe (n = 6).

Polarization

To assign mutation type (e.g., T**A**C > T**G**C), the ancestral allele state must first be determined. The software *mutyper* (DeWitt et al. 2023), which is used to assign mutation type, requires an ancestral genome fasta file in which the reference genome has been updated with ancestral states at polarized SNP positions (for example, if the reference genome contains an "A" at a particular site, but the ancestral state is determined to be "C", the ancestral fasta should have a "C" at that position).

Ancestral genome fasta files were previously generated for the human genome as part of the 1000 Genomes Project using the inferred great ape phylogeny at each site (Auton et al. 2015) (details in that paper's Supplement 8.3.1). Ancestral genome states were generated for the great ape species in Goldberg & Harris (2022) using parsimony. The remaining species had ancestral states assigned using *est-sfs* (Keightley and Jackson 2018), a program which takes SNPs from a focal species and one or more outgroups aligned to the same reference genome and uses parsimony and allele frequencies to estimate the probability that the major allele of the focal species is the ancestral state. Ancestral alleles can then be assigned probabilistically using a single draw from a binomial distribution per SNP, where the probability of success is the probability that the major allele is ancestral for the focal species: if the binomial draw yields '1' (success) then the major allele is assigned as likely to be ancestral; if it yields '0' then the minor allele is assigned as likely to be ancestral. Robinson et al. (2022) polarized the vaquita (*Phocoena sinus*; focal) in this manner with harbor porpoise (*Phocoena phocoena*; outgroup 1) and Indo-Pacific finless porpoise (*Neophocaena phocaenoides;* outgroup 2) as outgroups, and provided us with these previously unpublished data. We implemented *est-sfs* using the Kimura 2-parameter model and 10 maximum likelihood runs for the species in our dataset that had not been polarized by previous studies, using 1-2 outgroups depending on availability:

- polar bear (*U. maritimus*; focal), brown bear (*U. arctos*; outgroup 1), black bear (*U. americanus;* outgroup 2)
- brown bear (*U. arctos*; focal), polar bear (*U. maritimus*; outgroup 1), black bear (*U. americanus*; outgroup 2)
- Western European house mouse (*M. m. domesticus*; focal); southeastern Asian house mouse (*M. m. castaneus;* outgroup 1); Algerian mouse (*M. spretus;* outgroup 2)
- Algerian mouse (*M. spretus*; focal); Western European house mouse (*M. m. domesticus*; outgroup 1)
- Gray wolf (*C. lupus;* focal), coyote (*C. latrans;* outgroup1)
- Fin whale (*B. physalus;* focal), humpback whale (*Megaptera novaeangliae*; outgroup 1), blue whale (*Balaenoptera musculus*; outgroup 2)

Genome masking

To avoid regions under selection, regions that may be susceptible to poor genome quality calls due to repeat content, and regions known to have very atypical mutational processes compared to the rest of the genome, we generated mask files for every genome in order to mask:

1) exons ± 10kb on either side of each exon
2) repeat regions identified using *RepeatMasker* (Smit et al. 2013)
3) low-complexity regions identified using *Tandem Repeat Finder* (*TRF*) (Benson 1999)
4) CpG islands identified using *CpGPlot* (Larsen et al. 1992) or the UCSC pipeline (http://genome.ucsc.edu/cgi-bin/hgTrackUi?g=cpgIslandExt).

For most reference genomes in our study, the coordinates of these regions were available for download from the UCSC genome browser (https://genome.ucsc.edu/). For vaquita and fin whale, these masks are not available on the UCSC browser but were previously annotated and provided by the data generators (Robinson et al. (2022) and Nigenda-Morales et al. (2023), respectively). We generated the remaining files as follows: for the brown bear and polar bear we ran *RepeatMasker* (with species database set to "carnivore") (Smit et al. 2013). For polar bear, brown bear, and minke whale we ran *Tandem Repeat Finder* (*TRF*) (Benson 1999) to identify low-complexity regions using recommended parameters (and a maximum period size of 12 as in UCSC pipelines (http://genomewiki.ucsc.edu/index.php/TRF_Simple_Repeats) (parameters: match:2, mismatch: 5, delta: 7, PM: 80, PI: 10, minscore: 50, maxperiod: 12) and converted the output using a conversion script (https://github.com/Adamtaranto/TRF2GFF). Finally, for the brown bear, polar bear and minke whale genomes, we ran *EMBOSS CpGPlot* (Larsen et al. 1992) to identify CpG Islands using default parameters (window size: 100, minimum length of island: 200, minimum observed/expected ratio: 0.6, minimum percentage: 50).

We then combined all of these regions into one negative mask file in *.bed* format for each reference genome using *bedtools sort* and *bedtools merge* (Quinlan and Hall 2010). These regions were then masked out from all downstream analyses.

This masking approach is very conservative, masking 66-77% of the autosomal genome for each species. To ensure that our masking approach was sufficiently removing regions with potentially poor mappability, we compared the SNPs passing our masks to the *Umap* (Karimzadeh et al. 2018) mappability masks for the human (hg38) and mouse (mm10) reference genomes. When we applied the *Umap* Single Read (100bp) hg38 mask (download from the UCSC genome browser) to our filtered human SNPs, only 420 out of 2,121,078 SNPs were excluded, meaning that >99.9% of the SNPs passing our masks also pass the *Umap* mappability mask. We found similar results when we applied the *Umap* Single Read (100bp) mask for the mm10 mouse reference genome (downloaded from https://zenodo.org/record/60940) to our masked house mouse SNP dataset, with only 694 of 19,553,026 SNPs being excluded, resulting in >99.99% of our passing SNPs also passing the mouse mappability mask.

Based on these results, we concluded that our conservative masking approach is suitably excluding poorly-mapping regions for those species which do not have mappability masks.

Generating mutation spectra from polymorphism data

For each individual, we estimated a nested series of spectra at the 1-mer, 1-mer+CpG (CpG>TpG mutations as a separate category), 1-mer-minus-CpG (CpG>TpG mutations excluded), 3-mer, 5-mer and 7-mer levels from our polymorphism data, as described below.

Where sufficient individuals existed within a single population within a species, one population was chosen as representative for each species for downstream analyses: humans (African/AFR continental group), fin whale (Gulf of California population), and brown bear (ABC Islands population). First, to standardize sample sizes across datasets, five individuals were drawn at random from each population and used for all downstream analysis (**Table S1**). Sites that were missing genotypes across the selected individuals within a species were removed.

Each remaining variant's 7-mer mutation context (3bp on either side of each SNP) was extracted using the function *mutyper variants* (DeWitt et al. 2023), restricting to biallelic SNPs with no missing

data that are not fixed for the alternate allele and excluding the masked regions described above. In the human ancestral genome *.fasta* file, lower-case letters indicate sites with uncertain polarization; these were excluded using the --strict setting of *mutyper variants*. To enable the correction of these mutation spectra for the *k*-mer content of each species' reference genome, we also used the function *mutyper targets* to count the number of times each 7-mer was observed as part of each species' ancestral state *.fasta* file.

To generate individual-level mutation spectra we calculated each individual's 7-mer mutation spectrum (counts of each 7-mer mutation type) using the function *mutyper spectra*. SNPs with derived alleles appearing in more than one of these five individuals were randomly assigned to a single individual's mutation spectrum using the *–randomize* parameter to prevent shared variation from driving similarity between related individuals. These spectra were used to perform principal component analysis as described below.

To summarize the mutation spectrum of each population sample as a whole, we generated per-population spectra using *mutyper spectra* with the –population parameter, which is the equivalent of summing the randomized per-individual spectra.

To generate lower-dimensional mutation spectra, the 7-mer spectra generated per-individual and species described above were collapsed down into their 5-mer, 3-mer or 1-mer content (e.g., all 7-mers containing T**A**A>T**G**A as their central 3-mer were summed up to get the count of that mutation type for the 3-mer mutation spectrum).

See main **Methods** for how spectra were corrected for genome content and levels of genetic diversity.

Center log ratio (CLR) transformation of mutation spectrum data

The mutation spectrum is an example of a *compositional* data type, meaning that it is a distribution vector constrained to sum to 1. This internal dependency structure can cause downstream analyses of compositional data to be confounded by spurious correlations, but this problem can be largely eliminated by applying a geometrical transformation called the centered log ratio transform (CLR) that was developed by John Aitchison (Aitchison 1986).

We transformed our mutation spectrum data using the function *clr* from the *compositions R* package (van den Boogaart and Tolosana-Delgado 2008). The CLR transformation divides each value within the compositional spectrum by the geometric mean of the spectrum, then takes the natural logarithm. CLR-transformed spectra are used to compute the PCA and distance analyses described below. Because the Aitchison distance represents the difference between two logarithmic values, it makes comparisons between species' mutation counts proportional, instead of the absolute differences used in calculated un-transformed Euclidean distance. This has the additional advantage that variation in the most abundant mutation types does not dominate the differences between species to the degree that they can dominate distances between raw mutation spectrum vectors (**Figure S38**).

Since the CLR transformation is incompatible with mutation counts of zero, we regularized our data by adding a pseudocount of 1 to the number of mutations observed within each type category in each species. This regularization is done separately for 1-mer, 3-mer, 5-mer and 7-mer spectra. It contributes very little to 1-mer and 3-mer spectra since these have relatively high mutation counts per mutation type, even after downsampling, but is more critical for 5-mer and particularly 7-mer spectra which are sparser and therefore have many missing mutation types after downsampling.

We then converted the mutation counts to approximate 'rates' by dividing each count by the human-rescaled genomic target size of each *k*-mer, prior to CLR transformation.

An apparent limitation of this approach is that the final distances between species' mutation spectra will depend on the particular *k*-mer composition chosen for normalization (specifically, the human reference genome composition). However, we found that the CLR distance between mutation spectra is actually not dependent on this choice of *k*-mer composition as long as the species' spectra are rescaled to the same target sizes; an algebraic proof in **Note S2** shows that we could normalize our variant counts to any other nonzero *k*-mer distribution and obtain the same distance matrix. This proof also shows that as

long as the species' spectra are rescaled to the same genomic target composition, that same result would be obtained using mutation counts, mutation fractions, or mutation 'rates' (counts/targets) to represent the mutation spectrum (**Figure S39**). This implies that our results are not intrinsically sensitive to the genomic *k*-mer composition chosen to rescale all species' spectra to, nor to the particular set of species, genome assemblies or accessibility masks chosen for our study, except to the extent that they cause inclusion or exclusion of genomic regions with very different mutation spectra or systematic errors.

In addition to using the CLR, we also repeated our results using the ILR transform (isometric log-ratio) (Egozcue et al. 2003), with qualitatively similar results.

Principal component analysis

Principal component analysis was carried out on the per-individual mutation spectra (for the five randomly sampled individuals from each species) using *prcomp* in *R* with variables shifted to be 0-centered and scaled to have unit variance (scale = *T*, center = *T*). The PCA results were then plotted for every combination of PCs 1, 2 and 3 using *autoplot* from the *ggfortify R* package (Tang et al. 2016), coloring each point by its species identity. PCA loadings showing the contributions of each mutation type to the first two variance components are plotted below the main PCA figure.

We additionally plotted the PCA results coloring points by the sequencer used in each study, with points shaped as to whether 100bp paired-end or 150bp PE reads were used, to demonstrate that these qualitative variables do not explain the clustering seen in our data.

Aitchison distance (Euclidean distance of CLR-transformed vectors). Aitchison distance (the Euclidean distance between clr-transformed compositional vectors) was calculated between every pair of per-species spectra using the *dist* function in R.

Cosine similarity. We also measured cosine similarity between the non-transformed proportional mutation spectra, as this is a commonly used metric in cancer biology for comparing mutation signatures and spectra, using *sigfit's cosine_sim* function (Gori and Baez-Ortega 2020).

Phylogenetic distance

To calculate the phylogenetic distances between species, we used the maximum likelihood tree that Upham et al. (2019) generated using *RAxML* (Stamatakis 2014) from an alignment of 31 genes across 4,098 mammal species, including all the species included in our study. Upham et al. rooted the tree with *anolis* as its outgroup and constructed using the GTRCAT model within *RAxML*, with five independent replicates. The branch lengths of the *RAxML* tree represent the expected number of substitutions per site in the alignment. We subsetted the tree to include only our study species ("Balaenoptera_physalus" ,"Phocoena_sinus" ,"Ursus_maritimus" ,"Ursus_arctos" ,"Mus_musculus" ,"Mus_spretus","Homo_sapiens","Pongo_pygmaeus","Pongo_abelii","Pan_troglodytes","Pan_paniscus"," Gorilla_gorilla","Canis_lupus") using the *keep.tip* function in the R package *ape* (Paradis and Schliep 2019), and then calculated pairwise patristic distance (the sum of branch lengths between species) for every pair of species using the *cophenetic* function in *ape*. It is worth noting that the branch leading to the mouse species is very long (**Figure 2A**), a known feature of trees based on genetic distance that include rodents, likely due to their short generation times.

Additionally, we generated a second tree for our species using the tool *TimeTree* (Kumar et al. 2022) in which branch lengths represent divergence times between species scaled in millions of years (**Figure S1**). In this tree, the rodent branch is not longer than the other branches as the tree is scaled by time rather than genetic distance.

The function of these phylogenetic trees in our study is to determine the topology of the relationships among these species (which is not disputed) and the relative magnitude of their pairwise divergence for the Mantel test (below). We therefore chose to use state-of-the-art and highly cited pre-existing phylogenetic trees rather than generating new sequence alignments.

Mantel test for phylogenetic signal

Many methods, including Blomberg's *K* (Blomberg et al. 2003), have been developed to quantify phylogenetic signal: the degree to which a trait varies continuously across a phylogeny. However, few of them are capable of comparing high-dimensional traits, since they generally require each trait to be summarized by a single number at each tip of the phylogeny. Since each species' mutation spectrum is multidimensional and cannot be easily reduced to a single value, we are restricted to using a test for phylogenetic signal that is based on pairwise distances between tips. One test satisfying this constraint is the Mantel test for phylogenetic signal. The Mantel test calculates the correlation between two symmetrical distance matrices (one of pairwise distances in trait values, another of pairwise phylogenetic distances), and then permutes one matrix and recalculates the correlation. This is repeated for thousands of permutations, and a *p*-value is calculated based on the number of permutations which produce a correlation coefficient that equals or exceeds that of the real data. The Mantel test is underpowered compared to other tests for phylogenetic signal (Harmon and Glor 2010; Hardy and Pavoine 2012) but does not suffer from excess Type I error (Harmon and Glor 2010), even though the comparisons are not independent. It is important to note that the Mantel test, while an appropriate test for phylogenetic signal as performed here, should *not* be used in cases when two traits are being correlated without taking underlying phylogenetic signal into account (e.g. body weight and metabolism), as in that case it would suffer from excess Type I error (Harmon and Glor 2010; Guillot and Rousset 2013).

We tested for phylogenetic signal by quantifying the correlation between the pairwise Aitchison distance measured between species' mutation spectra and the square root of the pairwise phylogenetic distance calculated from either the RAxML tree (genetic distance) or the timetree (distance in evolutionary time). We used the square root of phylogenetic distance because under a Brownian motion model the trait distance is expected to scale linearly with the square root of phylogenetic distance (Hardy and Pavoine 2012).

We used the mantel function from the VEGAN (Dixon 2003) package in R to carry out the Mantel test. We calculated the Pearson correlation coefficient between genetic distance and mutation spectrum distance for each of 9,999,999 permutations. If no permutation yields a correlation coefficient higher than the correlation coefficient of the non-permuted data, VEGAN returns a *p*-value of 1 / (1+9,999,999) = 1e-7. We carried out a separate Mantel test for each mutation spectrum (1-mer, 1-mer+CpG, 1-mer-minus-CpG, 3-mer, 5-mer and 7-mer).

To confirm that our results were not due to the choice of a substitution-based tree, we repeated our analyses using cophenetic distances from the ultrametric time tree described above.
To confirm that our results were not due to how the data were polarized (ancestral states determined), we repeated the analysis using 'folded' mutation spectra, in which counts of reciprocal mutation types are added together (so that ACT > AAT and AAT>ACT mutations are not counted as separate categories, but are added together, meaning that mispolarization will not impact the results).

We also repeated our Mantel tests using a different distance measure between spectra: instead of Aitchison distance, we used the commonly-used metric cosine distance (1-cosine similarity) between spectra.

We additionally stratified the 3-mer, 5-mer and 7-mer spectra by central mutation type (treating all A>T *k*-mers separately from C>T *k*-mers when normalizing relative mutation rates, etc.) and carried out the Mantel test for distances calculated based on each central mutation type's *k*-mers. We set a significance threshold of $\alpha = 0.05/6$ for these tests to correct for multiple testing (6 central mutation types).

We also stratified the 3-mer, 5-mer and 7-mer spectra into three mutation categories depending on whether the ancestral and derived base pairs are "weak" (A:T) versus "strong" (G:C). This creates a class of weak-to-strong mutations that are positively selected by GC-biased gene conversion (A>C and A>G), a class of strong-to-weak mutations that are negatively selected (C>A and C>T), and a GC-conservative class that is neutrally evolving with respect to gBGC (A>T and C>G). We repeated the Mantel test within these stratified categories to verify that the mutation spectrum still exhibits

phylogenetic signal after removal of the component that might be the result of gBGC. We set the significance threshold as $\alpha = 0.05/3$ for these tests (three BGC categories).

In order to determine how much phylogenetic signal is contained in the 5-mer spectrum beyond what is driven by the phylogenetic signal of the central 3-mer, we randomized the counts of 5-mers within each central 3-mer category. We first stratified our 5-mer dataset by central 3-mer category (e.g. the AAA>ATA 3-mer category contains 16 5-mers: AAAAA>AATAA, TAAAA>TATAA, CAAAC>CATAC, etc.) and calculated the relative proportion of each of the 5-mers target size in the genome within a 3-mer category. We then carried out multinomial sampling to assign each of the total number of mutations in that 3-mer category to a 5-mer mutation type, using its relative target proportion in genome as its multinomial probability of occurring. Thus, mutations are assigned to 5-mers solely based on their relative *k*-mer frequency in the genome, not due to any underlying differences in mutation rate. This removes any possible phylogenetic signal that could exist due to differences in 5-mer mutation rate, with any residual phylogenetic signal driven by the phylogenetic signal of the 5-mers' central 3-mer. We generated 5,000 of these control spectra per species, then compared the distribution of Pearson's *r* values between these control spectra and phylogenetic distance with the *r* value of the empirical 5-mer spectrum. We calculated a *p*-value as [(the number of randomized control spectra with $r >=$ empirical) +1] / (5000 randomized datasets +1). We repeated this procedure for the 7-mer spectrum, randomizing 7-mer counts within each central 5-mer category.

To determine whether sparsity of 7-mers was driving the lack of phylogenetic signal beyond the 5-mer level, we repeated these analyses excluding the lowest-diversity species/populations in our dataset (removing the vaquita and polar bear and substituting the higher diversity Eastern North Pacific fin whales for the Gulf of California fin whales), resulting in the minimum number of SNPs in our dataset being ~890,000 instead of ~130,000. However, even with this higher number of SNPs (though reduced power due to fewer species), the 7-mer spectrum did not show a higher degree of phylogenetic signal than when randomized across 5-mer categories.

K_{mult} test for phylogenetic signal

We also tested for phylogenetic signal using a multivariate version of Blomberg's *K*, called K_{mult} (Adams 2014) as implemented in the *physignal* command in the R package *geomorph* (v. 4.0.5) (Adams et al. 2016; Baken et al. 2021) with 999 permutations.

Other possible sources of phylogenetic signal

We identified several bioinformatic "phenotypes" (such as genome assembly quality) that vary between species, as well as several biological phenotypes that are suspected to impact mutagenesis, and used the Mantel test to determine whether any of these confounders has as much phylogenetic signal as the mutation spectrum itself. We reasoned that any potential confounder that exhibits less phylogenetic signal than the mutation spectrum can be ruled out as a major driver of mutation spectrum variation between species.

To measure key bioinformatic phenotypes, we first calculated the average per-individual SNP sequence depth of the five individuals chosen at random from each species' dataset using *vcftools --depth* (Danecek et al. 2011) (the vaquita *.vcf* file did not contain depth information, so we used the average per-individual depth reported by Robinson et al. (2022)), and downloaded reference genome contig and scaffold N50 values from NCBI. To explore some biological phenotypes that might impact mutagenesis, we obtained estimates of age at first reproduction (in days) and reproductive lifespan (in days) for each species from (Pacifici et al. 2013). Vaquita was not included in that dataset and so we used estimates for harbor porpoise (*Phocoena phocoena*) as a proxy. Humans were also not included in that dataset, and so we added rough estimates of age at first reproduction and lifespan of age 22 years (8030 days) and ~18 years of reproductive lifespan (6570 days).

We then calculated the absolute value difference between every pair of species for each of these technical and biological phenotypes, and carried out the Mantel test to quantify correlation between these distances and phylogenetic distance (99,999 permutations). Note that fewer permutations were needed for

these tests than the Mantel tests for phylogenetic signal of the mutation spectrum, as no variable reached the *p*-value threshold (1-e5) that would have necessitated additional permutations.

We then used the Mantel test to directly measure correlation between these technical and biological confounding variables and the mutation spectrum, in both a phylogeny-unaware and phylogeny-aware manner. For the phylogenetically-unware manner (in which shared phylogenetic signal could contribute to a correlation between the variables and the mutation spectrum), we carried out the Mantel test as described above, with 99,999 permutations. For the phylogenetically-aware method, we used the *PhyloMantel* function from the *evolqg R* package (v. 0.2-9) (Melo et al. 2016), to carry out the Mantel test with phylogenetic permutations (in which more closely related species are more likely to be permuted).

Enrichment or depletion of particular *k*-mers

To look for lineage-specific enrichment or depletion of particular *k*-mer mutation types, we calculated the relative mutation rate of each mutation type within each species based on their original projected mutation spectrum counts (not rescaled, downsampled, or regularized). The relative rates were calculated as the rate of each particular *k*-mer (e.g. TTT**A**AAA>TTT**T**AAA count divided by the target size of TTTAAAA targets in the genome) divided by the rate of its central base pair mutation type (count of A>T mutations divided by total accessible A bases in the genome). The ratio of these two rates indicates whether a particular *k*-mer is enriched (ratio > 1) or depleted (ratio < 1) relative to its central 1-mer rate. We tested whether each of these ratios differs significantly from 1 using a two-sided Fisher's exact test (*fisher.test*() function in *R*) (this test was chosen over the Chi-Squared test because downsampled counts of some *k*-mers may be below 5). We then plotted the -log(10) *p*-values for each *k*-mer and looked for outlier *k*-mers that exceeded Bonferroni-corrected significance thresholds and had an enrichment that was even more extreme than each species' enrichment of CpG>TpG dimers.

To determine the impact of excluding CpG islands from our mutation spectra, we generated per-species mutation spectra within regions of the genome annotated as CpG islands and added this CpG island spectra to the spectra described above. Since CpG islands have a high CpG content, but a lower CpG>TpG mutation rate (Carlson et al. 2018), than other regions of the genome, this resulted in lower CpG>TpG enrichment rates over the background C>T rate for each species than when CpG islands are excluded. We report enrichment results for both sets of spectra (either with or without CpG islands included).

Investigating mouse-wolf spectra similarity

To confirm that the similarities we observe between the mouse and wolf spectra are not dataset specific or due to demographic history, we acquired two additional datasets: one dataset of mouse de novo mutation (DNM) 1-mer spectra (Lindsay et al. 2019) from whole genome sequencing of family trios, and a second wolf polymorphism dataset (Mooney et al. 2023) based on whole genome sequencing at the population level (which we call "wolf (UCLA)" as it was generated by a research group at that university, as opposed to "wolf (Broad)" which we used in our main analyses which was generated by the Broad Institute). We measured the Aitchison distance between each of these dataset's 1-mer spectra to those of all our other species datasets. The mouse DNM data had elevated Aitchison distance compared to all other interspecies comparisons, likely due to excess noise in the 1-mer spectrum due to sparse data, and/or to systemic difference between DNMs and polymorphisms. However, reassuringly, the closest polymorphism-based spectra to the mouse DNMs were our two mouse polymorphism datasets (house mouse and Algerian mouse), and remarkably, the next-closest were the two wolf datasets and the vaquita porpoise, confirming the similarities between these species' 1-mer spectra both at the DNM and polymorphism level, and across datasets.

Supplemental References

Adams DC. 2014. A Generalized K Statistic for Estimating Phylogenetic Signal from Shape and Other High-Dimensional Multivariate Data. *Syst. Biol.* [Internet] 63:685–697. Available from: https://doi.org/10.1093/sysbio/syu030

Adams DC, Collyer M, Kaliontzopoulou A, Sherratt E. 2016. Geomorph: Software for geometric morphometric analyses.

Adams DC, Collyer ML. 2019. Phylogenetic comparative methods and the evolution of multivariate phenotypes. *Annu. Rev. Ecol. Evol. Syst.* 50:405–425.

Aitchison J. 1986. The Statistical Analysis of Compositional Data. Springer Netherlands

Auton A, Abecasis GR, Altshuler DM, Durbin RM, Abecasis GR, Bentley DR, Chakravarti A, Clark AG, Donnelly P, Eichler EE, et al. 2015. A global reference for human genetic variation. *Nature* [Internet] 526:68–74. Available from: https://www.nature.com/articles/nature15393

Baken EK, Collyer ML, Kaliontzopoulou A, Adams DC. 2021. geomorph v4.0 and gmShiny: Enhanced analytics and a new graphical interface for a comprehensive morphometric experience. *Methods Ecol. Evol.* [Internet] 12:2355–2363. Available from: https://onlinelibrary.wiley.com/doi/abs/10.1111/2041-210X.13723

Barlow A, Cahill JA, Hartmann S, Theunert C, Xenikoudakis G, Fortes GG, Paijmans JLA, Rabeder G, Frischauf C, Grandal-d'Anglade A, et al. 2018. Partial genomic survival of cave bears in living brown bears. *Nat. Ecol. Evol.* [Internet] 2:1563–1570. Available from: https://www.nature.com/articles/s41559-018-0654-8

Benazzo A, Trucchi E, Cahill JA, Maisano Delser P, Mona S, Fumagalli M, Bunnefeld L, Cornetti L, Ghirotto S, Girardi M, et al. 2017. Survival and divergence in a small group: The extraordinary genomic history of the endangered Apennine brown bear stragglers. *Proc. Natl. Acad. Sci.* [Internet] 114:E9589–E9597. Available from: https://www.pnas.org/doi/full/10.1073/pnas.1707279114

Benson G. 1999. Tandem repeats finder: a program to analyze DNA sequences. *Nucleic Acids Res.* [Internet] 27:573–580. Available from: https://doi.org/10.1093/nar/27.2.573

Blomberg SP, Garland JR. T, Ives AR. 2003. Testing for Phylogenetic Signal in Comparative Data: Behavioral Traits Are More Labile. *Evolution* [Internet] 57:717–745. Available from: https://onlinelibrary.wiley.com/doi/abs/10.1111/j.0014-3820.2003.tb00285.x

van den Boogaart KG, Tolosana-Delgado R. 2008. "compositions": A unified R package to analyze compositional data. *Comput. Geosci.* [Internet] 34:320–338. Available from: https://www.sciencedirect.com/science/article/pii/S009830040700101X

Byrska-Bishop M, Evani US, Zhao X, Basile AO, Abel HJ, Regier AA, Corvelo A, Clarke WE, Musunuri R, Nagulapalli K, et al. 2022. High-coverage whole-genome sequencing of the expanded 1000 Genomes Project cohort including 602 trios. *Cell* [Internet] 185:3426-3440.e19. Available from: https://www.cell.com/cell/abstract/S0092-8674(22)00991-6

Cahill JA, Green RE, Fulton TL, Stiller M, Jay F, Ovsyanikov N, Salamzade R, John JS, Stirling I, Slatkin M, et al. 2013. Genomic Evidence for Island Population Conversion Resolves Conflicting Theories of Polar Bear Evolution. *PLOS Genet.* [Internet] 9:e1003345. Available from: https://journals.plos.org/plosgenetics/article?id=10.1371/journal.pgen.1003345

Carlson J, Locke AE, Flickinger M, Zawistowski M, Levy S, Myers RM, Boehnke M, Kang HM, Scott LJ, Li JZ, et al. 2018. Extremely rare variants reveal patterns of germline mutation rate heterogeneity in humans. *Nat. Commun.* [Internet] 9:3753. Available from: https://www.nature.com/articles/s41467-018-05936-5

Danecek P, Auton A, Abecasis G, Albers CA, Banks E, DePristo MA, Handsaker RE, Lunter G, Marth GT, Sherry ST, et al. 2011. The variant call format and VCFtools. *Bioinformatics* [Internet] 27:2156–2158. Available from: https://doi.org/10.1093/bioinformatics/btr330

DeWitt WS, Zhu L, Vollger MR, Goldberg ME, Talenti A, Beichman AC, Harris K. 2023. mutyper: assigning and summarizing mutation types for analyzing germline mutation spectra. *J. Open Source Softw.* [Internet] 8:5227. Available from: https://joss.theoj.org/papers/10.21105/joss.05227

Dixon P. 2003. VEGAN, a package of R functions for community ecology. *J. Veg. Sci.* [Internet] 14:927–930. Available from: https://onlinelibrary.wiley.com/doi/abs/10.1111/j.1654-1103.2003.tb02228.x

Egozcue JJ, Pawlowsky-Glahn V, Mateu-Figueras G, Barceló-Vidal C. 2003. Isometric Logratio Transformations for Compositional Data Analysis. *Math. Geol.* [Internet] 35:279–300. Available from: https://doi.org/10.1023/A:1023818214614

Goldberg ME, Harris K. 2022. Mutational Signatures of Replication Timing and Epigenetic Modification Persist through the Global Divergence of Mutation Spectra across the Great Ape Phylogeny. *Genome Biol. Evol.* [Internet] 14:evab104. Available from: https://doi.org/10.1093/gbe/evab104

Gori K, Baez-Ortega A. 2020. sigfit: flexible Bayesian inference of mutational signatures. :372896. Available from: https://www.biorxiv.org/content/10.1101/372896v2

Guillot G, Rousset F. 2013. Dismantling the Mantel tests. *Methods Ecol. Evol.* [Internet] 4:336–344. Available from: https://onlinelibrary.wiley.com/doi/abs/10.1111/2041-210x.12018

Hardy OJ, Pavoine S. 2012. Assessing phylogenetic signal with measurement error: a comparison of Mantel tests, Blomberg et al.'s K, and phylogenetic distograms. *Evol. Int. J. Org. Evol.* 66:2614–2621.

Harmon LJ, Glor RE. 2010. Poor statistical performance of the Mantel test in phylogenetic comparative analyses. *Evolution* [Internet] 64:2173–2178. Available from: https://doi.org/10.1111/j.1558-5646.2010.00973.x

Harr B, Karakoc E, Neme R, Teschke M, Pfeifle C, Pezer Ž, Babiker H, Linnenbrink M, Montero I, Scavetta R, et al. 2016. Genomic resources for wild populations of the house mouse, *Mus musculus* and its close relative *Mus spretus*. *Sci. Data* [Internet] 3:160075. Available from: https://www.nature.com/articles/sdata201675

Karimzadeh M, Ernst C, Kundaje A, Hoffman MM. 2018. Umap and Bismap: quantifying genome and methylome mappability. *Nucleic Acids Res.* [Internet] 46:e120. Available from: https://doi.org/10.1093/nar/gky677

Keightley PD, Jackson BC. 2018. Inferring the Probability of the Derived vs. the Ancestral Allelic State at a Polymorphic Site. *Genetics* [Internet] 209:897–906. Available from: https://doi.org/10.1534/genetics.118.301120

Kumar S, Suleski M, Craig JM, Kasprowicz AE, Sanderford M, Li M, Stecher G, Hedges SB. 2022. TimeTree 5: An Expanded Resource for Species Divergence Times. *Mol. Biol. Evol.* [Internet] 39:msac174. Available from: https://doi.org/10.1093/molbev/msac174

Larsen F, Gundersen G, Lopez R, Prydz H. 1992. CpG islands as gene markers in the human genome. *Genomics* [Internet] 13:1095–1107. Available from: https://www.sciencedirect.com/science/article/pii/088875439290024M

Lindsay SJ, Rahbari R, Kaplanis J, Keane T, Hurles ME. 2019. Similarities and differences in patterns of germline mutation between mice and humans. *Nat. Commun.* [Internet] 10:4053. Available from: https://www.nature.com/articles/s41467-019-12023-w

Liu S, Lorenzen ED, Fumagalli M, Li B, Harris K, Xiong Z, Zhou L, Korneliussen TS, Somel M, Babbitt C. 2014. Population genomics reveal recent speciation and rapid evolutionary adaptation in polar bears. *Cell* 157:785–794.

Melo D, Garcia G, Hubbe A, Assis AP, Marroig G. 2016. EvolQG - An R package for evolutionary quantitative genetics. *F1000Research* [Internet] 4:925. Available from: https://f1000research.com/articles/4-925/v3

Miller W, Schuster SC, Welch AJ, Ratan A, Bedoya-Reina OC, Zhao F, Kim HL, Burhans RC, Drautz DI, Wittekindt NE, et al. 2012. Polar and brown bear genomes reveal ancient admixture and demographic footprints of past climate change. *Proc. Natl. Acad. Sci.* [Internet] 109:E2382–E2390. Available from: https://www.pnas.org/doi/full/10.1073/pnas.1210506109

Mooney JA, Marsden CD, Yohannes A, Wayne RK, Lohmueller KE. 2023. Long-term Small Population Size, Deleterious Variation, and Altitude Adaptation in the Ethiopian Wolf, a Severely Endangered Canid. *Mol. Biol. Evol.* [Internet] 40:msac277. Available from: https://doi.org/10.1093/molbev/msac277

Morrill K, Hekman J, Li X, McClure J, Logan B, Goodman L, Gao M, Dong Y, Alonso M, Carmichael E, et al. 2022. Ancestry-inclusive dog genomics challenges popular breed stereotypes. *Science* [Internet] 376:eabk0639. Available from: https://www.science.org/doi/full/10.1126/science.abk0639

Nigenda-Morales SF, Lin M, Nuñez-Valencia PG, Kyriazis CC, Beichman AC, Robinson JA, Ragsdale AP, Urbán R. J, Archer FI, Viloria-Gómora L, et al. 2023. The genomic footprint of whaling and isolation in fin whale populations. *Nat. Commun.* [Internet] 14:5465. Available from: https://www.nature.com/articles/s41467-023-40052-z

Pacifici M, Santini L, Di Marco M, Baisero D, Francucci L, Marasini GG, Visconti P, Rondinini C. 2013. Generation length for mammals. *Nat. Conserv.* 5:89–94.

Paradis E, Schliep K. 2019. ape 5.0: an environment for modern phylogenetics and evolutionary analyses in R. *Bioinformatics* [Internet] 35:526–528. Available from: https://doi.org/10.1093/bioinformatics/bty633

Prado-Martinez J, Sudmant PH, Kidd JM, Li H, Kelley JL, Lorente-Galdos B, Veeramah KR, Woerner AE, O'Connor TD, Santpere G. 2013. Great ape genetic diversity and population history. *Nature* 499:471–475.

Quinlan AR, Hall IM. 2010. BEDTools: a flexible suite of utilities for comparing genomic features. *Bioinformatics* [Internet] 26:841–842. Available from: https://doi.org/10.1093/bioinformatics/btq033

Robinson JA, Kyriazis CC, Nigenda-Morales SF, Beichman AC, Rojas-Bracho L, Robertson KM, Fontaine MC, Wayne RK, Lohmueller KE, Taylor BL, et al. 2022. The critically endangered vaquita is not doomed to extinction by inbreeding depression. *Science* [Internet] 376:635–639. Available from: https://www.science.org/doi/full/10.1126/science.abm1742

Schubert M, Ermini L, Sarkissian CD, Jónsson H, Ginolhac A, Schaefer R, Martin MD, Fernández R, Kircher M, McCue M, et al. 2014. Characterization of ancient and modern genomes by SNP detection and phylogenomic and metagenomic analysis using PALEOMIX. *Nat. Protoc.* [Internet] 9:1056–1082. Available from: https://www.nature.com/articles/nprot.2014.063

Smit A, Hubley R, Green P. 2013. RepeatMasker 4.0. *Seattle WA Inst. Syst. Biol.*

Srivastava A, Kumar Sarsani V, Fiddes I, Sheehan SM, Seger RL, Barter ME, Neptune-Bear S, Lindqvist C, Korstanje R. 2019. Genome assembly and gene expression in the American black bear provides new insights into the renal response to hibernation. *DNA Res.* [Internet] 26:37–44. Available from: https://doi.org/10.1093/dnares/dsy036

Stamatakis A. 2014. RAxML version 8: a tool for phylogenetic analysis and post-analysis of large phylogenies. *Bioinformatics* [Internet] 30:1312–1313. Available from: https://doi.org/10.1093/bioinformatics/btu033

Tang Y, Horikoshi M, Li W. 2016. ggfortify: unified interface to visualize statistical results of popular R packages. *R J* 8:474.

Upham NS, Esselstyn JA, Jetz W. 2019. Inferring the mammal tree: Species-level sets of phylogenies for questions in ecology, evolution, and conservation. *PLOS Biol.* [Internet] 17:e3000494. Available from: https://journals.plos.org/plosbiology/article?id=10.1371/journal.pbio.3000494

Van der Auwera GA, Carneiro MO, Hartl C, Poplin R, Del Angel G, Levy-Moonshine A, Jordan T, Shakir K, Roazen D, Thibault J, et al. 2013. From FastQ data to high confidence variant calls: the Genome Analysis Toolkit best practices pipeline. *Curr. Protoc. Bioinforma.* 43:11.10.1-33.
